# Genomic determinants of sporulation in *Bacilli* and *Clostridia*: towards the minimal set of sporulation-specific genes

**DOI:** 10.1111/j.1462-2920.2012.02841.x

**Published:** 2012-11

**Authors:** Michael Y Galperin, Sergei L Mekhedov, Pere Puigbo, Sergey Smirnov, Yuri I Wolf, Daniel J Rigden

**Affiliations:** 1 National Center for Biotechnology Information, National Library of Medicine, National Institutes of HealthBethesda, MD 20894, USA; 2 Institute of Integrative Biology, University of LiverpoolCrown St., Liverpool L69 7ZB, UK

## Abstract

Three classes of low-G+C Gram-positive bacteria (*Firmicutes*), *Bacilli*, *Clostridia* and *Negativicutes*, include numerous members that are capable of producing heat-resistant endospores. Spore-forming firmicutes include many environmentally important organisms, such as insect pathogens and cellulose-degrading industrial strains, as well as human pathogens responsible for such diseases as anthrax, botulism, gas gangrene and tetanus. In the best-studied model organism *Bacillus subtilis*, sporulation involves over 500 genes, many of which are conserved among other bacilli and clostridia. This work aimed to define the genomic requirements for sporulation through an analysis of the presence of sporulation genes in various firmicutes, including those with smaller genomes than *B. subtilis*. Cultivable spore-formers were found to have genomes larger than 2300 kb and encompass over 2150 protein-coding genes of which 60 are orthologues of genes that are apparently essential for sporulation in *B. subtilis*. Clostridial spore-formers lack, among others, *spoIIB*, *sda*, *spoVID* and *safA* genes and have non-orthologous displacements of *spoIIQ and spoIVFA*, suggesting substantial differences between bacilli and clostridia in the engulfment and spore coat formation steps. Many *B. subtilis* sporulation genes, particularly those encoding small acid-soluble spore proteins and spore coat proteins, were found only in the family *Bacillaceae*, or even in a subset of *Bacillus* spp. Phylogenetic profiles of sporulation genes, compiled in this work, confirm the presence of a common sporulation gene core, but also illuminate the diversity of the sporulation processes within various lineages. These profiles should help further experimental studies of uncharacterized widespread sporulation genes, which would ultimately allow delineation of the minimal set(s) of sporulation-specific genes in *Bacilli* and *Clostridia*.

## Introduction

Three classes of low-G+C Gram-positive bacteria (*Firmicutes*), *Bacilli*, *Clostridia* and *Negativicutes*, include numerous members capable of producing endospores that show dramatically increased resistance to a variety of environmental challenges, such as heat, solvents, oxidizing agents, lysozyme, UV irradiation and desiccation (Setlow, 2007). Sporulation is an important survival mechanism that allows spore-forming firmicutes to withstand adverse environmental conditions and spread around the earth and potentially even in outer space (Setlow, 2007; Horneck *et al*., 2010). In addition, the recently noted ‘eat resistance’ refers to the ability of spore-formers to resist predation by protozoa (Klobutcher *et al*., 2006) and might also be important for their persistence in gastrointestinal tracts of various animals, from insects to human (Hong *et al*., 2009). Some spore-formers are important pathogens that cause anthrax, food poisoning, infectious diarrhoea, colitis, gas gangrene, tetanus and other diseases, whereas others are important environmental microorganisms that are being used for pest control, wood processing, fuel production and more (Jensen *et al*., 2003; Rasko *et al*., 2005; Dürre, 2008; Peck, 2009).

Sporulation is tightly linked to cell division and shares with it a number of regulatory checkpoints (Veening *et al*., 2009). In the best-studied model organism *Bacillus subtilis*, sporulation affects expression of more than 500 genes, acting in a highly regulated manner (Piggot and Losick, 2002; Eichenberger *et al*., 2003; 2004; Piggot and Hilbert, 2004; Steil *et al*., 2005; Wang *et al*., 2006). Compendia of genes that are involved in sporulation of *B. subtilis* have been compiled through (i) studies of asporogenousmutants, (ii) identification of genes whose expression depends upon the master regulator of sporulation, Spo0A, and sporulation-specific sigma factors σ^E^ and σ^K^ (in the mother cell) or σ^F^ and σ^G^ (in the developing spore), (iii) proteomic analysis of the spore content, and most recently and (iv) RNA-seq profiling of sporulation gene expression (Eichenberger *et al*., 2003; 2004; Lai *et al*., 2003; Molle *et al*., 2003; Liu *et al*., 2004; Steil *et al*., 2005; Bergman *et al*., 2006; Wang *et al*., 2006; Lawley *et al*., 2009; Mao *et al*., 2011). Sporulation genes are typically characterized by the timing of expression, from stage 0 to stage VI, in addition to – or in lieu of – their known or putative biochemical functions. Functional assignments of many sporulation genes are based solely on the phenotypes of the respective mutations (sporulation arrest at a certain stage or production of immature spores) and their products still remain to be characterized with respect to their enzymatic activity, if any, protein–protein interactions, ligand binding and/or three-dimensional structure.

Despite the medical, environmental and industrial importance of many spore-formers, studies of sporulation mechanisms have been mostly limited to *B. subtilis*, *Bacillus anthracis* and their closest relatives. There have been relatively few studies on sporulation in *Clostridium acetobutylicum, Clostridium difficile* and *Clostridium perfringens* (Alsaker and Papoutsakis, 2005; Paredes *et al*., 2005; Jones *et al*., 2008; Lawley *et al*., 2009; Underwood *et al*., 2009; Steiner *et al*., 2011) and even fewer on sporulation in other bacteria. As a result, information on the sporulation genes of firmicutes, other than *B. subtilis*, *B. anthracis* or *C. acetobutylicum*, has been obtained primarily by genome sequence analysis.

In 2002, Stragier analysed the distribution of 66 sporulation genes among the five firmicute genomes available at that time (*B. subtilis*, *B. anthracis*, *Bacillus stearothermophilus*, *C. acetobutylicum* and *C. difficile*) and classified those genes into six groups based on their presence in (i) all spore-formers, (ii) some *Bacillus* and some *Clostridium* spp., (iii) all *Bacillus* spp. but not *Clostridium* spp., (iv) some *Bacillus* spp. and no *Clostridium* spp., (v) some *Clostridium* spp. but not *Bacillus* spp. and (vi) only *B. subtilis* (Stragier, 2002). The following year, Eichenberger and colleagues (2003) characterized the σ^E^ regulon of *B. subtilis* and tested the presence of the identified genes in the same five-genome set with the addition of *Oceanobacillus iheyensis*; genomes of non-spore-formers *Listeria monocytogenes* and *L. innocua* were used as negative control (Eichenberger *et al*., 2003). The same approach has been applied in two subsequent studies that analysed sporulation gene expression in the mother cell and the forespore (Eichenberger *et al*., 2004; Wang *et al*., 2006). In 2004, Wiegel and co-workers examined sporulation genes in 12 bacillar and 5 clostridial genomes and used PCR and hybridization techniques to identify four tell-tale sporulation genes (*spo0A*, *sspA* and *dpaAB*) in a variety of firmicutes (Onyenwoke *et al*., 2004). This study has introduced the important distinction between asporogenous (non-spore-forming) firmicutes which encode few, if any, sporulation genes and non-sporogenous (or ‘conditionally non-spore-forming’) bacteria that have close spore-forming relatives, encode a large number of sporulation genes and have lost the ability to form spores owing to only a few (relatively recent) mutations (Onyenwoke *et al*., 2004). Several subsequent reports on sequencing of various firmicute genomes included detailed analyses of the presence of *B. subtilis* sporulation genes in the respective genomes (Wu *et al*., 2005; Chivian *et al*., 2008; Lawley *et al*., 2009). Most recently, de Hoon and colleagues traced the presence of 511 *B. subtilis* sporulation-related genes in the genomes of 24 firmicute species, including 12 genomes of bacilli and 12 genomes of various clostridia (de Hoon *et al*., 2010), while Xiao and colleagues (2011) analysed the distribution in *Bacilli* and *Clostridia* of known and putative germination-related genes. Unfortunately, genome descriptions of many firmicutes do not mention whether the respective strains are able to form spores (Nonaka *et al*., 2006; Pierce *et al*., 2008). Genomes of some firmicutes have been sequenced without formally describing the organisms, so that information on their ability to form spores is still unavailable (Byrne-Bailey *et al*., 2010).

The phylogenetic distribution of sporulation-specific genes of *B. subtilis* (i.e. those genes whose expression depends on Spo0A and/or sporulation sigma factors) proved to be quite complex, with many of them missing in certain bacillar and clostridial genomes (Onyenwoke *et al*., 2004; Wu *et al*., 2005; Rigden and Galperin, 2008). Such genes appeared to be non-essential for spore formation, perhaps playing regulatory roles. Conversely, close homologues of some *B*. *subtilis* sporulation genes have been identified outside of the *Firmicutes*, for example, in the genomes of certain cyanobacteria, proteobacteria and spirochaetes (Onyenwoke *et al*., 2004; Rigden and Galperin, 2008). Such genes typically encode cell division proteins, enzymes of peptidoglycan turnover, transcriptional regulators or components of bacterial signal transduction systems. Recent studies of the sporulation signalling networks revealed major differences between bacilli and clostridia and even among various bacilli (de Hoon *et al*., 2010; Steiner *et al*., 2011). However, in most cases, comparative genome analyses have aimed at characterization of the regulation of the sporulation process and relatively less effort – with the exception of the work of Stragier (2002) and Wu and colleagues (2005) – has been devoted to defining the minimal set of sporulation genes, i.e. the set of genes that are necessary and sufficient for producing a viable heat-resistant spore. Such genes – roughly defined as those whose mutation decreases spore formation by at least an order of magnitude – appear to constitute only a relatively small fraction of all genes whose expression is stimulated by sporulation. Further, because of the presence of multiple paralogues and alternative regulatory pathways, some genes become essential for sporulation only in a certain mutant background. Thus, defining the list of genes that are essential for sporulation remains a non-trivial but a potentially useful task.

In the past several years, over 100 complete genomes of spore-forming *Firmicutes* have been sequenced. Therefore we reasoned that a comparative study of the sporulation genes identified in bacillar and clostridial genomes could be helpful for sorting out the sets of essential and auxiliary sporulation-specific genes, identifying likely cases of non-orthologous gene displacement and getting an insight into the evolution of sporulation. Here, we present the results of a comprehensive study of the distribution of sporulation-specific genes and employ these data to analyse the (in)ability of certain bacterial species to form mature spores. We also present novel functional predictions for some uncharacterized proteins involved in sporulation. We hope that phylogenetic profiles of the distribution of sporulation genes, compiled in this work and available on the website http://www.ncbi.nlm.nih.gov/Complete_Genomes/Sporulation.html, will stimulate experimental studies aimed at determining the functions of uncharacterized widespread sporulation genes and will help in delineation of the minimal set(s) of sporulation-specific genes in *Bacilli* and *Clostridia*.

## Results

### Correlation of sporulation with phylogeny and genome size

**Fig. 1:**
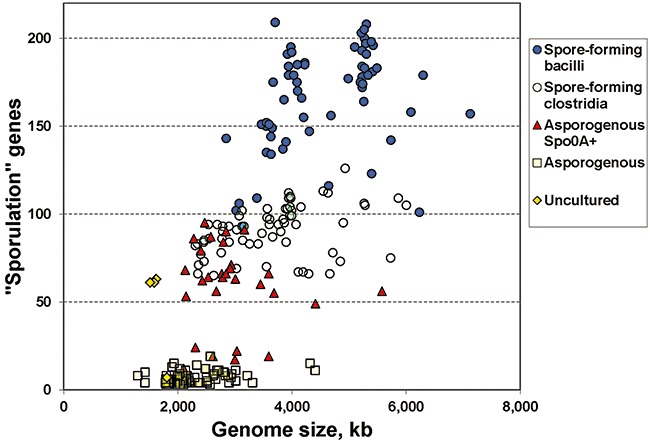
Distribution of ‘sporulation’ genes in the genomes of *Firmicutes*. The plot shows the number of proteins encoded in whose annotation includes the words ‘spore’ or ‘sporulation’. Dark blue circles, spore-forming members of *Bacilli*; light green circles, spore-forming members of *Clostridia*; triangles, asporogenous bacteria that encode Spo0A; squares, asporogenous bacteria that do not encode Spo0A; diamonds, uncultured *Candidatus* Arthromitus spp. and *Clostridiales* genomospecies BVAB3.

By the end of 2011, the list of completely sequenced firmicute genomes has grown to almost 400 (Pruitt *et al*., 2012), with 141 of these genomes coming from 83 known or likely spore-forming species (see Table S1). Our initial sorting of all these genomes into those of spore-forming and asporogenous bacteria was based on the presence of the sporulation master regulator Spo0A, a transcriptional response regulator with a unique DNA-binding output domain (Lewis *et al*., 2000) that has never been detected outside the *Firmicutes* phylum (Galperin, 2006). The genomes were also examined for the presence of three other sporulation genes (*sspA* and *dpaAB*) previously used by Onyenwoke and colleagues (2004) to judge the ability of bacteria to form spores. However, neither the presence of any one of these genes nor even their combination could be used as a clear-cut predictor of the organism's ability to form spores as all four genes are present in some bacteria known to be non-sporogenous, such as *Caldicellulosiruptor* spp*.* and *Natranaerobius thermophilus* (Table S2). Conversely, the *dpaA* and *dpaB* genes are missing in several well-known spore-forming clostridia, such as *C. acetobutylicum, C. botulinum, C. kluyveri* and *C. perfringens* (Table S1), in accordance with previous observations (Onyenwoke *et al*., 2004; Orsburn *et al*., 2010). To supplement the distribution patterns of these four genes, we calculated the number of genes whose annotation included the words ‘spore’ or ‘sporulation’ encoded in each firmicute genome. Finally, we checked whether the initial microbiological descriptions of the sequenced strains (or the respective genera) contained clear indications of their ability – or inability – to form spores. While bacilli generally had more proteins annotated as involved in sporulation than clostridia (Fig. 1), most firmicutes fell into two major categories: (i) spore-forming bacteria that encoded Spo0A and at least 60 ‘sporulation’ genes and (ii) asporogenous bacteria with no Spo0A and fewer than 15 ‘sporulation’ genes. However, these analyses also revealed 30 genomes of apparently non-spore-forming bacteria (Table S2) that encoded Spo0A and from seven (*Clostridiales* genomospecies BVAB3) to 91 (*N. thermophilus*) putative ‘sporulation’ genes (Fig. 1).

The first category includes well-characterized spore-formers *Bacillus* spp., *Clostridium* spp. as well as *Alkaliphilus* spp., *Desulfotomaculum* spp., *Thermoanaerobacter* spp. and several other genera. It also includes three recently sequenced genomes of unculturable segmented filamentous bacteria *Candidatus* Arthromitus spp. (Kuwahara *et al*., 2011; Prakash *et al*., 2011; Sczesnak *et al*., 2011). Although *Cand.* Arthromitus spp. have not been cultured so far, their spores have been observed by electron microscopy and found to be viable after treatment with 3% chloroform (Chase and Erlandsen, 1976; Kuwahara *et al*., 2011). The second category includes asporogenous Gram-positive bacteria, such as lactic acid bacteria, listeria, staphylococci, streptococci and other genera. An example of the third category is *Macrococcus caseolyticus*, which until recently has been assigned to the genus *Staphylococcus* and definitely does not produce spores (Kloos *et al*., 1998). This organism encodes a typical Spo0A protein (Table S2) but very few other sporulation proteins (just Spo0M, SpoVB, SpoVG, Jag), and even those are not unique for spore-formers (Rigden and Galperin, 2008). This category also includes such organisms as the aforementioned *N. thermophilus* or *Caldicellulosiruptor* spp*.*, which have not been observed to form spores (Rainey *et al*., 1994; Bredholt *et al*., 1999; Mesbah *et al*., 2007; Miroshnichenko *et al*., 2008a) but nevertheless encode numerous sporulation genes (Blumer-Schuette *et al*., 2011; Zhao *et al*., 2011). Although the possibility remains that (some of) these bacteria simply have not been cultured under conditions that would force them to form spores, most of them appeared to lack one or more genes essential for sporulation. Thus, one of the goals of this work was the identification of the minimal set of genes that are essential for sporulation.

Spore-forming firmicutes typically have larger genomes than their asporogenous counterparts and we found no (cultured) spore-formers with genome sizes of less than 2300 kb (Fig. 1). The only exceptions were the aforementioned reduced genomes (1516–1620 kb) of unculturable *Cand.* Arthromitus spp. (Kuwahara *et al*., 2011; Prakash *et al*., 2011; Sczesnak *et al*., 2011). Most of the non-spore-forming firmicutes, such as lactobacilli, staphylococci and streptococci, have smaller genomes, although members of the *Butyrivibrio*, *Eubacterium* and *Oscillibacter* genera are asporogenous despite having relatively large genome sizes (Fig. S1).

Analysis of the phylogenetic distribution of the spore-formers showed that the ability to form spores is widespread in the classes *Bacilli* and *Clostridia* although certain groups within these classes are entirely devoid of spore-forming representatives (Table 1). There were no spore-formers with completely sequenced genomes in the two other classes of *Firmicutes*, *Erysipelotrichi* and *Negativicutes*[the latter class includes the spore-forming genera *Sporomusa* and *Acetonema* (Möller *et al*., 1984; Tocheva *et al*., 2011)], or among the *Mollicutes*[recently reclassified into a separate phylum *Tenericutes* (Ludwig *et al*., 2009)]. In some cases, non-spore-formers are nested within spore-forming lineages and could be attributed to the loss of certain sporulation genes. Thus, *Bacillus selenitireducens* (3592 kb) and *Clostridium sticklandii* (2715 kb) have the smallest genomes among the members of the respective genera, carry the smallest number of sporulation genes and are unable to form spores (Switzer Blum *et al*., 1998; Fonknechten *et al*., 2010).

**Table 1:** Distribution of spore-forming bacteria among *Firmicutes*

Class, order^a^	Family^a^	Complete genomes, species^b^	Spore-formers in the set	Non-sporogenic members of spore-forming clades (examples)
***Bacilli***				
*Bacillales*	*Bacillaceae*	29	28	*Bacillus selenitireducens*
	*Listeriaceae*	5	None	
	*Paenibacillaceae*	7	7	
	*Staphylococcaceae*	8	None	
	Other	5	3	*Exiguobacterium sibiricum*
*Lactobacillales*	*Lactobacillaceae*	21	None	
	*Leuconostocaceae*	7	None	
	*Streptococcaceae*	18	None	
	Other	5	None	
***Clostridia***				
*Clostridiales*	*Clostridiaceae*	19	17	*Clostridium* sp. SY8519^c^
	*Eubacteriaceae*	3	None	
	*Peptococcaceae*	12	11	*Filifactor alocis*
	Other	16	6	*Finegoldia magna*
*Halanaerobiales*	*Halobacteroidaceae*	4	None	
*Thermoanaero bacterales*	*Thermoanaerobacte raceae*	12	10	*Ammonifex degensii*
	*Family III Incertae Sedis*	8	None	
	Other	6	4	*Coprothermobacter proteolyticus*^d^
***Erysipelotrichi***	*Erysipelotrichaceae*	1	None	
***Negativicutes***	*Veillonellaceae, Acidaminococcaceae*	4	None	
***Mollicutes***^a^	*Acholeplasmataceae, Mycoplasmataceae*	27	None	

a. Taxonomy is according to the NCBI Taxonomy database (Federhen, 2012) and the ribosomal proteins-based tree (Ciccarelli *et al*., 2006; Yutin *et al*., 2012), which are generally consistent with the Bergey's Taxonomic Outline (Ludwig *et al*., 2009). *Negativicutes* have been recently recognized as a separate class (Marchandin *et al*., 2010), whereas *Mollicutes* were re-classified into a separate phylum *Tenericutes* (Ludwig *et al*., 2009). See Table S1 for the complete list.

b. As of the end of 2011; based on a non-redundant set that includes a single representative genome for each individual species.

c. The second genome of non-sporulating member of *Clostridiaceae* is that of *C. tetani* E88, a non-sporulating variant of strain Massachusetts used in vaccine production (Brüggemann *et al*., 2003).

d. Placing of *Coprothermobacter proteolyticus* within *Clostridia* is not supported by either ribosomal protein-based phylogeny (Yutin *et al*., 2012) or whole-genome analysis (Beiko, 2011; Nishida *et al*., 2011).

With the exception of *M. caseolyticus* and *Cand.* Arthromitus spp., the smallest genome sizes among Spo0A-encoding bacteria are found in the clostridial family *Thermoanaerobacteraceae* which includes both spore-forming and non-sporogenous bacteria. Of the 12 members of *Thermoanaerobacteraceae* with sequenced genomes, 11 appear to form spores whereas *Ammonifex degensii* that has the smallest (2157 kb) genome in the family is a non-spore-former (Huber *et al*., 1996). Remarkably, its close relative *Ammonifex thiophilus* is capable of forming spores (Miroshnichenko *et al*., 2008b). *Thermoanaerobacter mathranii*, which has the second smallest (2306 kb) genome in the family, has been shown to form spores (Larsen *et al*., 1997). Other *Thermoanaerobacter* spp. whose genome sizes range from 2345 to 2457 kb either have been shown to form spores or have been predicted to do so (Hemme *et al*., 2010). Thus, there appears to be a clear correlation between genome size and the ability to sporulate and the genome sizes of *Thermoanaerobacteraceae* mark a clear boundary between free-living spore-formers and non-spore-formers at ∼ 2200–2300 kb (Fig. S1B).

In the class *Bacilli*, all spore-forming members belong to the order *Bacillales* (Table 1) and typically have genome sizes greater than 3.2 Mb. The only exception is *Anoxybacillus flavithermus* (Saw *et al*., 2008), whose 2847 kb genome is the smallest among the non-clostridial spore-formers. With the exception of some spore coat-encoding genes, *A. flavithermus* carries the full set of genes that are thought to be important for sporulation of *B. subtilis* (see below and Table S3).

### The sporulation-specific gene set

In *B. subtilis and C. acetobutylicum* sporulation affects expression of numerous genes (Fawcett *et al*., 2000; Eichenberger *et al*., 2003; 2004; Molle *et al*., 2003; Steil *et al*., 2005; Wang *et al*., 2006; Jones *et al*., 2008), not all of which are necessarily involved in spore formation. Indeed, the developing spore contains the full genetic complement of the vegetative cell. Genetic and proteomic screens of the mother cell and the forespore have detected expression of genes for ribosomal proteins, cell division proteins, various metabolic enzymes and other housekeeping genes (Fawcett *et al*., 2000; Molle *et al*., 2003; Jones *et al*., 2008; Lawley *et al*., 2009) that might be important for sporulation but also function in the vegetative cell. Such genes were not considered sporulation-specific and therefore have been excluded from the analysed set. Because of that, with the single exception of the peptidyl-tRNA hydrolase SpoVC (Moran *et al*., 1980; Menez *et al*., 2002), no genes in this set (Table S3) were essential for the vegetative growth of *B. subtilis* (Kobayashi *et al*., 2003). The resulting set contained 651 genes that have been shown to be preferentially (or exclusively) expressed during sporulation (see Table S3). Only a relatively small fraction of these genes appeared to be essential for sporulation and many had no characterized biochemical function (Table S3).

### Reliability of the phylogenomic patterns

The analysis of the patterns of phylogenetic distribution of sporulation genes in this work relied on the COG approach, used in the well-known Clusters of Orthologous Groups of proteins (COG) database (Tatusov *et al*., 1997; 2000), as modified in subsequent studies (Mulkidjanian *et al*., 2006; Makarova *et al*., 2007). Briefly, proteins encoded in the selected firmicute genomes were assigned to the existing set of COGs (http://www.ncbi.nlm.nih.gov/COG/) and the remaining sporulation proteins were unified in clusters based on their bidirectional best blastp hits. Under this approach, the claim that a particular gene is present in a given genome means only that there is an open reading frame (ORF) whose protein product can be assigned to the respective COG. This does not necessarily imply that the protein in question is functional: it might lack key amino acid residues or even domains and as a result could lack the expected activity. Conversely, the statement that a certain gene is absent from certain genome means only that this genome lacks an ORF whose product would be assignable to the given COG. The COG method does not rely on arbitrary cut-offs in assessing protein sequence similarity and has previously proven to be sufficiently robust in identifying highly divergent orthologous genes (Natale *et al*., 2000; Tatusov *et al*., 2000; Makarova *et al*., 2007). Nevertheless, there is always a possibility that a sequence has diverged too far from the general consensus to be recognized as a member of the given family. For low-complexity proteins, such as those found in the spore coat, recognition of a conserved sequence motif, if any such exists, becomes particularly complicated. The resulting protein clusters were manually inspected to validate their phylogenetic patterns; COGs containing short ORFs, which are often overlooked in genome annotation, and potentially mistranslated widespread genes were checked using the tblastn (Altschul *et al*., 1997) searches against the respective genomes. The missed ORFs identified in this manner were submitted to the RefSeq database (Pruitt *et al*., 2012); these ORFs are highlighted in green in Table S3 and a partial list is presented in Table 2.

**Table 2:** Widespread sporulation genes omitted in genome annotation

		Newly identified genes		
			
Gene	Protein size^a^	No.	Identified in genomes^b^	Corrected phylogenetic distribution^c^
*bofA*	87	2	*Paenibacillus polymyxa E681, Clostridium tetani E88*	All *Bacillales*, most clostridia
*cotD*	75	4	*Geobacillus kaustophilus, G.* *thermodenitrificans*	Some *Bacillaceae*
*safA*	387	1	*Bacillus thuringiensis BMB171*	All *Bacillaceae*
*sda*	52	6	*B.* *thuringiensis, O.* *iheyensis*	Most *Bacillales*
*spmB*	178	1	*Paenibacillus* sp. *Y412MC10*	All spore-formers
*spo0B*	192	1	*Paenibacillus* sp. *Y412MC10*	All *Bacillales*
*spoIIIAC*	68	1	*Bacillus thuringiensis BMB171*	All spore-formers
*spoIIIAF*	206		*Paenibacillus polymyxa E681*	All spore-formers
*spoIVA*	492	1	*Clostridium cellulolyticum H10*	All spore-formers
*spoVAEA*	203	4	*B.* *anthracis, B.* *cereus*	All *Bacillaceae*
*spoVM*	26	70	*B.* *anthracis,* *B. cereus, C.* *difficile, C.* *botulinum, S.* *thermophilum*	All *Bacillales*, most clostridia
*sspK*	50	5	*B.* *cytotoxicus, B.* *thuringiensis*	All *Bacillaceae*
*sspL*	42	3	*O.* *iheyensis, G.* *thermodenitrificans*	Some *Bacillaceae*
*sspM*	34	17	*B.* *megaterium, B.* *thuringiensis*	Most *Bacillaceae*
*sspN*	48	4	*B.* *clausii, B.* *thuringiensis*	All *Bacillaceae*
*sspO*	48	3	*B.* *cereus, B.* *thuringiensis*	Most *Bacillaceae*
*sspP*	48	9	*B.* *clausii, B.* *thuringiensis, Paenibacillus* sp. *Y412MC10*	All *Bacillaceae*
*tlp*	83	1	*Clostridium tetani E88*	Most *Bacillales*, some clostridia
*yabP*	100	1	*Clostridium tetani E88*	All spore-formers

a. Length (amino acid residues) of the respective protein from *Bacillus subtilis* strain 168.

b. Bacterial genera are abbreviated as follows: *B., Bacillus; C., Clostridium; G, Geobacillus; O., Oceanobacillus., S., Symbiobacterium.*

c. Phylogenetic distribution among spore-forming clades; ‘*Bacillales*’ indicates members of families *Bacillaceae, Alicyclobacillaceae* and *Paenibacillaceae*, except for *B. selenitireducens* and *Exiguobacterium* spp. (see Table 1).

Despite the efforts to refine the sporulation-specific protein set, certain genes that are believed to be essential for sporulation of *B. subtilis* were not found in the genomes of some known spore-formers (Tables 3 and S3). By far the largest number of such missing (as opposed to mistranslated) genes was in the genome of *Lysinibacillus sphaericus* C3-41 (Hu *et al*., 2008). Despite the relatively large size (4817 kb) and known ability of *L. sphaericus* C3-41 to form spores (Hu *et al*., 2008), the genome of this bacterium lacks *bofC*, *gerM*, *spmA*, *spmB*, *sda*, *spoIIB*, *spoIIM*, *spoIIIAA*, *spoIIIAB*, *spoIIIAD*, *spoIIIAF*, *spoVAA*, *spoVAB*, *spoVID*, *tlp* and *yqfC* genes and has frameshifts in *obgE*, *spoIVA*, *spoIVB* and *spoIVFB* genes (Table S3). Because *spmAB*, *spoIIM* and the full set of *spoIIIA* genes are essential for sporulation of *B. subtilis* and are found in the genomes of all other spore-formers (Table 3), the genome sequence of *L. sphaericus* C3-41 was deemed insufficiently reliable and was not used to judge whether certain missing genes are dispensable for sporulation. However, some of the same genes (*gerM*, *sda*, *spoIIB*, *spoVID*, *spo0M*) were also missing in the genomes of both members of the family *Alicyclobacillaceae*, *Alicyclobacillus acidocaldarius* and *Kyrpidia* (formerly *Bacillus*) *tusciae* (Table S3), which could be attributed to (i) their phylogenetic distance from *Bacillaceae* and *Paenibacillaceae* and (ii) their somewhat smaller genome sizes [3206 and 3385 kb respectively (Chen *et al*., 2011; Klenk *et al*., 2011)] than other spore-forming bacilli (Fig. S1). As a result, *gerM*, *sda*, *spoIIB*, *spoVID* and *spo0M* genes were assumed to be dispensable for sporulation. Subsequent phylogenetic analysis revealed the absence in the *A. acidocaldarius* genome of such widespread genes as *spoIIP*, *spoVAA*, *spoVAB*, *tcyA* and *degV* (*yviA*), which are all present in *K. tusciae*. Owing to the uncertainty whether these differences stem from the smaller genome size of the *A. acidocaldarius* genome or represent sequencing errors, genes that were missing only in a single genome in the analysed set were still included in Table 3.

**Table 3:** Sporulation genes conserved in bacilli and clostridia

	Phylogenetic distribution of the genes		
	
Sporulation stage	All spore-forming bacilli and clostridia	All bacilli and most clostridia	Most bacilli, some clostridia
Stage 0 (pre-septation)	***spo0A, sigH (spo0H)***^b^, *spo0J, **obgE***	*spo0E*^a^, *rapA(spo0L) family*^a^^,^^c^, *yjcM, ylbF, yyaA*	*spo0M, spo0F, ytxC*
Stage II (post-septation)	***spoIIAA**, spoIIAB, **sigF (spoIIAC)***^b^*, **spoIID**, spoIIE (spoIIH)*, ***spoIIGA, sigE* (*spoIIGB*)*, spoIIM, spoIIP***^a^*, **spoIIR***		
Stages III-VI (post-engulfment)	***cwlD***^a^*, **dacB, dapA, dapB, spmA, spmB, spoIIIAA, spoIIIAB, spoIIIAC, spoIIIAD, spoIIIAE, spoIIIAF***^a^*, **spoIIIAG, spoIIIAH, spoIIID***^a^*, **spoIIIE, spoIIIJ**, jag*^a^*, **sigG (spoIIIG), spoIVA, spoIVB, sigK (spoIIIC+spoIVCB)**, spoVAC, spoVAD*^a^, *spoVAEB, **spoVB** family, **pth (spoVC), spoVD***^a^*, **spoVG***^a^, ***spoVK***^a^*, **spoVS***^a^, *spoVT, **stoA (spoIVH), yabP, yabQ***^a^*, **ylbJ**, ylmC, **yqfC, yqfD**, ytvI, yyaC*	***bofA, spoIVFB**, spoVAEA, spoVAF, **spoVE, dpaA (spoVFA), dpaB (spoVFB)***^b^*, **ald (spoVN), spoVR**, sspA* family, *ydcC, yhbH, yqfU, **ytrH, yunB***	*spoVAA, spoVAB, yfhM, ykuD, ypqA, yqfS, ytrI*
Spore coat	***spoIVA**, alr (yncD)*	***spoVM**, cotJC, cotF* family, *lipC* (*ycsK*), *yaaH*^a^, *yabG*^a^, *ydhD*^a^, *yhaX, yhbA, yhbB, yhcN, yhcQ, yhjR, yjqC*	*cotA, cotC, cotH, cotI, cotJA, cotJB, cotM, cotP, cotS, cotU, tgl, yisY, yknT*
Germination	*gpr, lgt (gerF)*	*gerA* family, *gerB* family, *gerC* family, *gerM, ypeB, ytgP*	

Genes that appear to be essential for sporulation of *B. subtilis* are shown in bold typeface.

a. These genes are missing in one or two genomes because of a frameshift or a possible sequencing error.

b. Gene names in parentheses indicate alternative names of the same genes.

c. The *cotF* family includes *cotF, yhcQ, yraD, yraF* and *yusN* genes; *gerA* family includes *gerAA, gerBA, gerKA, yfkQ* and *yndD* genes; *gerB* family includes *gerAB, gerBB, gerKB, gerXB, yfkT* and *yndE* genes; *gerC* family includes *gerAC, gerBC, gerKC, yfkR* and *yndF* genes; *rapA* family includes *rapA, rapB, rapC, rapD, rapE, rapF, rapG, rapH, rapI, rapJ* and *rapK; spoVB family includes spoVB, ykvU* and *ytgP; sspA* family includes *sspA, sspB, sspC* and *sspD* genes.

Similarly to the genomes of the two *Alicyclobacillaceae*, the five genomes of the members of the family *Paenibacillaceae* showed similar patterns of presence and absence of sporulation genes (Table S3). Phyletic patterns of the four *Paenibacillus* spp. were most similar to each other, while *Brevibacillus brevis,* the fifth member of the family, had a more divergent phyletic pattern (Table S3). These findings point to a general correlation between the taxonomic proximity of the organisms and similarity of their phyletic patterns (which therefore could be referred to as phylogenetic patterns). Indeed, these patterns appeared to be consistent among closely related bacteria, such as the *Bacillus cereus* group; the *B. subtilis* group; the *Bacillus halodurans–B. pseudofirmus–B. clausii* cluster; the *Paenibacillaceae*; the *Thermoanaerobacteraceae*; the *C. acetobutylicum*–*beijerinckii*–*botulinum*–*perfringens* group, and other tight groups (Fig. S2), but not necessarily between these groups. In the end, for the purposes of this work, interpretation of the phylogenetic patterns was performed with the caveat that the absence of a particular gene in a single genome (or two closely related genomes coming from the same sequencing centre) does not necessarily imply that this gene is non-essential for sporulation (see Table 3).

### Conservation of the core sporulation genes among *Bacilli* and *Clostridia*

Previous studies have demonstrated conservation of the core sporulation pathway within *Bacilli* (*B. subtilis*, *B. anthracis*) and between *Bacilli* and *Clostridia* (*C. acetobutylicum*, *C. difficile*) (Stragier, 2002; Paredes *et al*., 2005; Lawley *et al*., 2009; de Hoon *et al*., 2010). Indeed, phylogenetic profiling showed that most of the sporulation genes included in the category 1 of the Stragier list (Stragier, 2002) are conserved in all spore-formers (Table 3). The presence of these genes in spore-forming bacteria with dramatically different lifestyles and relatively small genome sizes, including *Thermoanaerobacter* spp. and *Cand*. Arthromitus spp. (see below), suggests that the set of genes that are conserved in all currently available spore-former genomes (Table 3) represents a close approximation of the true minimal set of sporulation-specific genes. However, because functions of many sporulation proteins remain unknown, we could not properly account for the cases of non-orthologous gene displacement, whereby the same (e.g. essential for sporulation) function in different organisms is carried out by proteins belonging to two or more distinct protein families. The specific case of the likely non-orthologous gene displacement of SpoIIQ in clostridia is discussed below but there might be other similar cases.

Although many proteins that are known to be essential for sporulation of *B. subtilis* are also conserved among the spore-forming clostridia (Table 3), there are substantial differences between bacillar and clostridial spore-formers. One of such differences is the previously noted direct phosphorylation of Spo0A by clostridial sporulation sensor kinases without the involvement of the Spo0F–Spo0B–Spo0A phosphorelay (Worner *et al*., 2006; Steiner *et al*., 2011). Other key differences include the absence in clostridia of orthologues of such bacillar genes as *spoIIB*, *spoIIQ*, *spoIVFA*; many genes encoding small acid-soluble spore proteins (SASPs); genes encoding morphogenetic proteins SpoVID, Sda, CotE and CotZ, which are involved in spore coat assembly; and many other spore coat proteins (Tables 4 and S3). In addition, many sporulation genes that are widespread in bacilli are found only in a handful of clostridia (Table S3). Some of these discrepancies warrant further scrutiny. Below, we discuss the substantial differences between the two groups in the regulation of the onset of sporulation, the engulfment process and the assembly of the spore coat.

**Table 4:** *Bacilli*-specific sporulation genes

	Phylogenetic distribution of the genes	
	
Sporulation stage	All bacilli, no clostridia	Most bacilli, no clostridia
Stage 0	*spo0B*	*kinA, kinB, kinD, kinE, kbaA, sda*
Stage II	*spoIIQ*	*spoIIB, sirA (yneE)*
Stages III–VI	*spoIVFA, yqhG*	*nucB, sspE, sspK, sspM, sspN, sspO, sspP, ybaK, ycgG, yfhD, yfhS, yfkD, yjbA, yjcA, ylbE, yneF, yozQ, ypfB, ypjB, yppF, ypzA, yqfT, yqfX, yqfZ, yqhP, yrrS, yteV, ytxG, ywrJ*
Spore coat		*spoVID, safA, spoVIF, cotB, cotD, cotN (tasA), cotO, cotY/cotZ,* c*oxA, yeeK, ylbD, ymaG, ypeP, yppG, ypzA, ysxE, yutH, yxeE*
Unassigned		*yppE, ywjG*

Phylogenetic profiles of *B. subtilis* sporulation genes that are found primarily within the class *Bacilli* also show a clear separation between the core and auxiliary genes (Table S3). Most genes that are essential for *B. subtilis* sporulation are conserved throughout the family *Bacillaceae* and, with several exceptions discussed above, also in *Alicyclobacillaceae* and *Paenibacillaceae* (Table S3). In contrast to this core set, there was considerable diversity among the genes encoding SASPs, spore coat proteins, spore coat polysaccharide biosynthesis proteins and spore germination proteins: although every bacillar genome encoded at least some of those, their exact content varied even between closely related organisms (Table S3).

### Spo0A∼P regulatory cascade

In both bacilli and clostridia, the key regulatory switch that launches the sporulation process is phosphorylation of the transcriptional response regulator Spo0A, which leads to its dimerization and dramatically increases its affinity to its target sites on the DNA (Lewis *et al*., 2002). In *B. subtilis*, Spo0A phosphorylation can be triggered by any of the five sensor histidine kinases, sporulation-specific sensor kinases KinA, KinB, KinC, KinD or KinE and reversed by aspartate phosphatases Spo0E, YnzD and YisI (Perego, 2001). The phosphorylation cascade from the sporulation kinases to Spo0A goes through the response regulator Spo0F and the phosphotransferase Spo0B and is subject to complex regulation, which includes response regulator aspartate phosphatases (encoded by 11 paralogous genes named from *rapA* to *rapK*), the short peptides that are co-transcribed with these phosphatases and render them inactive (7 annotated peptides from PhrA to PhrK), as well as transcriptional regulators of their expression and oligopeptide transporters that regulate availability of the inhibitory peptides. Studies of clostridial sporulation revealed the absence in *C. acetobutylicum, C. botulinum* or *C. difficile* of clear orthologues of the sporulation sensor kinases KinA-KinE, as well as of Spo0B, Spo0F and Spo0E (Worner *et al*., 2006; Steiner *et al*., 2011). Instead, in clostridia, Spo0A can be directly phosphorylated by several distinct sensor histidine kinases (CBO1120 in *C. botulinum*, CD1579 and CD2492 in *C. difficile*, CAC0323, CAC0903 and CAC3319 in *C. acetobutylicum*). Like the sporulation-specific histidine kinases KinA–KinD of *B. subtilis*, each of these clostridial histidine kinases, except for CBO1120, contains a ligand-binding PAS domain and activity-related HisKA and HATPase domains but they otherwise share little sequence similarity with bacillar sporulation kinases, particularly in their sensory N-terminal region (Worner *et al*., 2006; Underwood *et al*., 2009; Steiner *et al*., 2011). Direct phosphorylation of Spo0A in clostridia also shows up in the absence of the phosphorelay proteins Spo0B (Table 4) and Spo0F, as well as the sporulation control protein Spo0M (Table S3). In fact, several clostridial genomes (e.g. in the family *Peptococcaceae*) encode single-domain response regulators of the CheY/Spo0F family (Galperin, 2010) that appear more closely related to Spo0F than to CheY (Table S3) but, in the absence of Spo0B, they are likely to have alternative, non-sporulation-related functions. In summary, clostridia seem to encode a streamlined version of the Spo0A phosphorylation pathway with fewer components and fewer checkpoints than bacilli.

### Engulfment

In *B. subtilis*, the engulfment process is driven by the interaction of membrane-associated proteins on both sides of the mother cell–forespore interface: the eight-protein SpoIIIA complex on the mother cell side and the membrane-anchored protein SpoIIQ on the forespore side of this interface (Blaylock *et al*., 2004; Doan *et al*., 2005; 2009; Aung *et al*., 2007; Campo *et al*., 2008). The SpoIIIA–SpoIIQ complex is believed to anchor the serine phosphatase SpoIIE and proteins SpoIID, SpoIIM, SpoIIP and BofA, which are further required for proper localization of SpoIVFA (Doan *et al*., 2005; Campo *et al*., 2008). All these proteins are found in all spore-forming bacilli, indicating that the SpoIIIA–SpoIIQ ‘zipper’ is a common feature of bacillar sporulation. However, while all eight proteins of the SpoIIIA complex (from SpoIIIAA to SpoIIIAH), SpoIIE, SpoIID, SpoIIP and SpoIIM are encoded in all spore-formers (Table 2), there are no orthologues of SpoIIQ or SpoIVFA in any clostridia (Table 3).

SpoIIQ and SpoIVFA both contain Zn-dependent peptidase M23-like (LytM) domains, which are likely to be catalytically inactive owing to the amino acid substitutions in their active sites (see Fig. S3 and Meisner and Moran, 2011). In SpoIIQ, this LytM domain is responsible for the localization of SpoIIQ to the mother cell–forespore interface (Meisner and Moran, 2011). In clostridia, the positions equivalent to *spoIIQ* and *spoIVFA* genes are occupied by non-orthologous genes that encode proteins combining the same LytM domain with other, apparently unrelated, N-terminal domains. For example, *C. difficile* gene *CD0125* is located between *spoIID* and *spoIIID* genes and also encodes an apparently inactive (Fig. S3) membrane-anchored LytM domain. Stragier (2002) referred to this protein as ‘clostridial SpoIIQ’, while mentioning its distant relationship to bacillar SpoIIQ proteins. Recent structural studies of SpoIIQ–SpoIIIAH interaction in *B. subtilis* identified the region of SpoIIQ that is responsible for its interaction with SpoIIIAH (Levdikov *et al*., 2012; Meisner *et al*., 2012). This very short (15 aa) region, consisting of an α-helix (α1) and two β-strands (β2–β3), forms an insertion in the typical LytM domain structure, suggesting that the differences in the N-terminal domains of SpoIIQ and CD0125 families do not preclude them from carrying out the same function. Indeed, our analysis found orthologues of CD0125 encoded in nearly all spore-forming members of families *Clostridiaceae* and *Thermoanaerobacteraceae* (Table S3). However, no orthologues of *CD0125* were found in the genomes of *Carboxydothermus hydrogenoformans*, *Moorella thermoacetica*, *Natranaerobius thermophilus*, or in spore-forming members of *Peptococcaceae*, such as *Cand*. Desulforudis audaxviator, *Desulfitobacterium hafniense* or *Desulfitobacterium* spp. (Table S3). Thus, while there is a definite possibility that the CD0125 family proteins – or other LytM-domain proteins – indeed function as non-orthologous gene displacements of SpoIIQ in some clostridia, there are several organisms for which such replacement proteins still remain to be identified. Alternatively, engulfment in (some) clostridia could proceed without SpoIIQ, as has been shown in *spoIIQ*^-^ mutants of *B. subtilis* (Sun *et al*., 2000; Chiba *et al*., 2007). It would definitely be interesting to learn which clostridial proteins, if any, interact with SpoIIIA.

Further, while *spoIID*, *spoIIM* and *spoIIP* are found in all spore-forming bacilli and clostridia (*spoIIP* appears to be absent in *A. acidocaldarius*), *spoIIB* and *spoIVFA* have not been found in any clostridia (Table 3). Thus, clostridia seem to be missing both localization pathways [SpoIIB-dependent and SpoIVFA-dependent (Aung *et al*., 2007)] that could guide SpoIID, SpoIIM and SpoIIP proteins to the division septum. The absence of SpoIVFA also suggests that clostridia employ distinct mechanisms of regulation of pro-σ^K^ processing. Remarkably, *C. difficile* and *C. saccharolyticum* are also missing *spoIVFB* and *bofA* genes (Table S1), which is probably related to the absence of pro-σ^K^ processing in *C. difficile* (Haraldsen and Sonenshein, 2003).

### Spore core

Early descriptions of the spore core noted the presence of conventional cellular proteins as well as certain spore-specific proteins (Spudich and Kornberg, 1968; Singh *et al*., 1977). Recent proteomic analyses of the spore contents confirmed the presence of ribosomal proteins, metabolic enzymes, chaperones and other housekeeping proteins (Lawley *et al*., 2009). However, a significant fraction of soluble proteins (up to 20% of the total spore protein of *B. subtilis*) consists of SASPs, whose molecular weights range from 7 to 12 kD (Setlow, 1975; Johnson and Tipper, 1981). Transcription of the SASP genes is dependent on the sporulation-specific sigma factor σ^G^; these proteins bind DNA and participate in its protection against heat, UV radiation and other damaging agents (Setlow, 1988; 2007; Driks, 2002).

*Bacillus subtilis* encodes 16 SASP types named from SspA to SspP and two additional ones, Tlp and CsgA (Driks, 2002). Three of these proteins, SspA, SspB and SspE, are most abundant in its spores and correspond to the major SASP bands on the CM-cellulose chromatography column (termed alpha, beta and gamma respectively (Setlow, 1975; Johnson and Tipper, 1981). Two of these SASPs, SspA and SspB, are very similar in sequence and form the alpha/beta group, which also includes less abundant (minor) SASPs SspC, SspD and SspF. These five proteins share significant sequence similarity (Setlow, 2007) and, with the exception of SspF, their homologues in other firmicute genomes could not be readily assigned to a particular subfamily. As a result, SspA, SspB, SspC and SspD were all mapped into a single COG, whereas SspF could be assigned to a different COG. Some clostridia also encode an SspF-related form of the α/β group protein, referred to as Ssp4 (Li and McClane, 2008). Alpha/beta class SASPs form a multigene family (Fliss *et al*., 1986; Setlow, 2007) with the five genes of *B. subtilis* placing it in the middle of the range seen in spore-forming *Firmicutes*: the abundance of these genes ranges from two in *A. flavithermus*, *Cand*. Desulforudis and *Cand*. Arthromitus to 7–8 in various strains of *B. cereus* and 12 in the genome of *C. beijerinckii* (Table S3). Remarkably, most bacilli carry multiple paralogues of *sspA* (*sspA*-*sspD*) and only a single copy of *sspF*. In contrast, clostridia typically carry multiple copies of *sspF* and either a single copy of the *sspA* gene (members of the genus *Thermoanaerobacter*) or none at all (all other clostridia). The same pattern, a single *sspA* and multiple copies of *sspF*, is seen in four *Paenibacillus* spp.; *K. tusciae* carries four copies of *sspF* but its single *sspA* gene is disrupted by a frameshift.

Aside from SASPs of the α/β group, most *B. subtilis* SASPs have a relatively narrow phylogenetic distribution and are found almost exclusively in *Bacilli*. Thus, the major SASP of the γ-type, SspE, is encoded in most bacilli but is absent in any clostridial genome sequenced to date (Table S3 and Vyas *et al*., 2011). Among minor SASPs, only SspH, SspI and Tlp are found in any clostridia, although each of these three is found in almost all bacilli. The first, SspH, has a patchy distribution in clostridia; for example, it is encoded in *Alkaliphilus metalliredigens* but not in closely related *Alkaliphilus oremlandii*. There are two copies of the *sspH* gene in the most strains of *C. botulinum*, a single copy in *C. acetobutylicum* and *C. kluyveri*, and none in *C. difficile*, *C. perfringens* and most other clostridia. The Tlp SASP has a similarly patchy distribution in clostridia, whereas the SspI protein is encoded in almost every bacillar genome but absent in all clostridia except for the members of *Thermoanaerobacterales*. Finally, minor SASPs SspG, SspJ, SspK, SspL, SspM, SspN, SspO and SspP are found only a small number of bacilli.

The total number of SASP genes in spore-forming bacilli is fairly constant and ranges from 11 in *B. clausii* to 22 in *Bacillus megaterium*. The only exceptions are *L. sphaericus* with seven genes and the two members of *Alicyclobacillaceae* with five and six genes respectively (Table S3). Several clostridial genomes carry just two SASP genes, *ssp4* and/or *sspF* (Table S3). The highest number of SASP genes among clostridia is 14 (12 α/β-type, *sspH* and *tlp*), found in *C. beijerinckii* (Table S3).

Taken together, these data indicate that formation of viable spores does not require a great diversity of SASPs. The SASP genes are easily duplicated, forming multigene families (Fliss *et al*., 1986), and easily lost; for example, *B. selenitireducens* does not encode any SASPs. On the other hand, some asporogenous clostridia encode multiple SASPs: each of the *Caldicellulosiruptor* spp. carries three paralogous copies of the *sspF* gene; *A. degensii* and *Halothermothrix orenii* have four of them. Obviously, evolution of this gene family was quite complex and included multiple tandem duplications and a likely gene loss. The presence of these genes in asporogenous bacteria probably reflects a relatively recent loss of sporulation by these organisms. Alternatively, it might indicate that protection from DNA damage afforded by SASPs was a beneficial trait that could be preserved even after the loss of sporulation.

### Spore cortex

The peptidoglycan layer that surrounds the (inner) forespore membrane is referred to as the spore cortex (Popham, 2002). In *B. subtilis*, genes believed to be involved in the formation of the spore cortex, *spoVB*, *spoVD*, *spoVE*, *yabP*, *yabQ*, *ylbJ*, *yqfC* and *yqfD*, are transcribed in the mother cell compartment under the direction of the sigma factor σ^E^ (Fawcett *et al*., 2000; Asai *et al*., 2001; Eichenberger *et al*., 2003). All these genes appear to be essential for the formation of mature spores [with the possible exception of *yabP* (Liu *et al*., 2010)] and, accordingly, each of them is found in all spore-forming firmicutes (Table 2), demonstrating a remarkable conservation of the spore cortex biosynthesis.

Upon germination, spore cortex peptidoglycan is hydrolysed by a joint action of several widely conserved cortex-lytic enzymes, including SleB, SleL (YaaH) and YpeB. In addition. some clostridia encode SleC, which combines lytic transglycosylase and *N*-acetylmuramoyl-l-alanine amidase activities (Kumazawa *et al*., 2007) and is absent in bacilli (Table S3).

### Spore coat

The layers of the spore shell surrounding the outer membrane are collectively referred to as the spore coat. According to the recent studies, assembly of the *B. subtilis* spore coat depends on the SpoIVA protein, which is recruited to the forespore membrane by SpoVM (McKenney *et al*., 2010; McKenney and Eichenberger, 2011). In turn, SpoIVA interacts with SpoVID, SafA, CotE, LipC (YcsK), YhaX, YheD, YjzB and YppG, forming the base layer of the spore coat, after which the SafA- and CotE-interacting proteins form the inner and outer spore coat respectively. Remarkably, of these 10 proteins, only SpoIVA is universally present in all bacilli and clostridia (Table S3). SpoVM is a very short (< 30 aa) protein that has been rarely recognized in genome annotation. We were able to identify the *spoVM* gene in the genomes of all bacilli and some, albeit not all, clostridia (Table S3 and Fig. S4). It remains to be seen whether SpoVM plays the same role in clostridia as it does in bacilli and whether distant versions of SpoVM are encoded in *Alkaliphilus* spp., *Clostridium phytofermentans*, *C. saccharolyticum*, *Thermoanaerobacter* spp. and other genomes where we were unable to find it through standard database searches. All bacillar spore-formers encode CotE and all except for *A. acidocaldarius* and *K. tusciae* encode SpoIVD and SafA (Table S3), suggesting that the mechanisms of assembly of the spore coat are shared by (nearly) all bacilli. On the other hand, we have not seen CotE, SpoIVD and SafA encoded in clostridia, indicating substantial differences from bacillar spore coat assembly in clostridia (and also in the members of *Alicyclobacillaceae*). Most other *B. subtilis* spore coat proteins have narrow phylogenetic distribution, with *yjzB*, *cotT*, *cotG*, *cotQ*, *cotR*, *cotSA*, cotV, *cotW*, cotX, *oxdD*, *yraE*, *yraG*, *ytxO*, *ywqH*, *yuzC*, *yxeF* and *yybI* genes missing in most members of the *B. cereus* group (Table S3). *Anoxybacillus flavithermus*, with its relatively small genome size, additionally lacks *cotA*, *cotB*, *cotF*, *cotH*, *cotJA*, *cotJB*, *cotN*, *cotO*, *cotT*, cotY, *yheD*, *yodI* and *yeeK* genes (Table S3). These observations show that mature spores could be formed with a much smaller set of coat proteins than the one described in *B. subtilis* (Driks, 2002; Imamura *et al*., 2011; McKenney and Eichenberger, 2011). Further, *cgeAB* and *cgeCDE* operons encoding ‘spore coat maturation proteins’[components of the outermost spore layer (Imamura *et al*., 2011)] are only found in the *B. subtilis* group; they are absent in *B. cereus* group and in other bacilli and clostridia (Table S3). Conversely, certain components of the exosporium are limited to the members of the *B. cereus* group and are missing in the *B. subtilis* group and in other bacteria.

In general, genes for most coat proteins exhibit complex phyletic patterns that do not necessarily correlate with the phylogenetic proximity of the host organisms. These patterns probably reflect a complex evolutionary history of the respective bacteria, driven by specific ecological adaptations, including antigenic divergence of the spore coats of host-associated organisms. An interesting example of such complex phyletic patterns is the distribution of transglutaminase (Tgl), an enzyme implicated in ε-(γ-glutamyl) lysine isopeptide cross-linking of GerQ molecules at the late stages of spore maturation (Ragkousi and Setlow, 2004; Zilhão *et al*., 2005). *Tgl*-like genes have been detected in the genomes of *B. subtilis* and several other *Bacillus* spp. but not in the genomes of non-spore-forming bacteria, which indicated a specific role in sporulation (Zilhão *et al*., 2005). Our work showed that, indeed, Tgl is encoded in the majority of bacilli and in just two clostridial species, *C. botulinum* (most strains) and *C. kluyveri* (Table S3). However, in accordance with the observation that cross-linking of GerQ (and potentially of other spore coat proteins), catalysed by this enzyme, is not essential for the spore formation or their stability (Ragkousi and Setlow, 2004), *tgl* gene is missing in *Anoxybacillus*, *Oceanobacillus*, *Bacillus cellulosilyticus* and several other bacilli.

Summing up, distribution of *B. subtilis* spore coat proteins among other bacillar and clostridial spore-formers probably reflects distinctive adaptations of these organisms to their specific ecological niches. Spore coats of other firmicutes are likely to contain additional, still unidentified, proteins; potential candidates include several families of low-complexity proteins, identified in this work (Table S3).

### Improved annotation of sporulation genes

As noted above, despite extensive studies of the sporulation process, many sporulation genes remain poorly characterized with respect to their molecular functions. The existing annotations based on locus designations and relating to their roles in sporulation often give a somewhat misleading impression as to the extent of current understanding of the biochemical activities of the respective proteins, which in many cases remain unknown (Rigden and Galperin, 2008). Even among the widespread genes that appear essential for sporulation (Table 3), there appears to be no data on the enzymatic activity (if any) and no structural characterization of products of *spo0M*, *spmA*, *spmB*, *spoIIM*, *spoIIP*, *spoIIR* and many other genes (Table S3).

In order to improve functional annotation of the sporulation genes included in the present compilation, we compared the respective protein sequences against public domain databases, such as Pfam, CDD, COGs, InterPro and TIGRFAMs (Tatusov *et al*., 2000; Selengut *et al*., 2007; Marchler-Bauer *et al*., 2011; Hunter *et al*., 2012; Punta *et al*., 2012) and included protein family-based biochemical annotation, wherever possible, into Table S3.

As an example, sequence analysis of the so-called response regulator aspartate phosphatases RapA–RapK revealed that they all consist of the tetratricopeptide repeat (TPR) domain that is normally devoid of any enzymatic activity. Therefore, these proteins apparently do not have a phosphatase activity of their own; rather, their binding to Spo0F∼P seems to activate the intrinsic autophosphatase activity of Spo0F, in accordance with the previous observations (Tzeng *et al*., 1998). Therefore, RapA-RapK proteins are referred to in Table S3 as ‘Spo0F∼P-binding proteins’, rather than ‘aspartate phosphatases’.

We also applied remote homology detection tools to discover functionally informative but non-trivial evolutionary relationships. In most cases, there were no helpful homologies, as sporulation proteins either mapped into their own separate families or showed distant relationships to known sequence families or determined structures but the similarities were too subtle to indicate a functional relationship (D.J.R., unpubl. obs.). In several cases, however, newly discovered distant sequence similarity was supported by the conservation of known catalytic residues, which improved confidence in predicted enzymatic functions (Table S4). Thus, CotH, a broadly distributed protein found also in deltaproteobacteria, actinobacteria and other non-spore-formers (Rigden and Galperin, 2008), was identified as a likely protein kinase, YhcO as a metalloprotease, YngK as a glycoside hydrolase, and YhbB and YndL as (possibly peptidoglycan degrading) amidases (Table S4). These predictions have clear biological implications, suggesting, e.g. involvement of protein phosphorylation in regulation of the spore coat assembly, carried out by the CotH protein (Naclerio *et al*., 1996; Zilhão *et al*., 2004; Isticato *et al*., 2008) and a possible involvement of YndL in cleavage of the y-glutamate links between spore coat proteins, created by the transglutaminase (see above).

These activities reflect the general trends among poorly characterized sporulation proteins, whose deduced enzymatic activities were predominantly hydrolytic (glycoside or peptidoglycan hydrolases) with an addition of some glycoside transferases (Table S3). Other sporulation proteins appeared to have either regulatory or protein-binding (or peptidoglycan-binding) function. The codon adaptation values of sporulation proteins, presented in Table S3, show that many of them could be highly expressed (at a certain stage of sporulation), making them priority targets for experimental studies.

A important feature of spore proteome is the presence of multiple proteins with predicted ‘house-cleaning’ activities that purge the cell from potentially harmful compounds (Galperin *et al*., 2006). These include systems for detoxification of arsenate (ArsB and ArsC) and oxygen and various ROS compounds (catalase, superoxide dismutase, peroxiredoxin, thiol peroxidase and alkyl hydroperoxide reductase), spore photoproduct (thymine dimer) lyase and pyrophosphatases of NUDIX (MutT) and MazG superfamilies that hydrolyse non-canonical NTPs (Moroz *et al*., 2005). Remarkably, superoxide dismutase and other oxygen detoxification proteins are widespread among the strictly anaerobic clostridia, suggesting that the presence of these genes represents a specific adaptation, beneficial for long-term survival of spores and not just a stress response system as it is often described.

### Properties of non-sporogenous strains

Using the set of the widely conserved sporulation genes, presented in Table 3, it becomes possible to explain the properties of at least some organisms that encode Spo0A but still do not form viable spores (category 3 on Fig. 1). Table 5 lists some of such species and the widely conserved sporulation genes that are missing in their genomes. It shows that while some Spo0A^+^ bacteria lack a significant number of sporulation genes (cf. Fig. 1), others do not seem to miss any (known) essential genes; their apparent inability to form viable spores could be due to point mutations in those genes and/or to certain combinations of deletions of otherwise non-essential genes.

**Table 5:** Examples of apparently essential sporulation genes missing in Spo0A^+^ non-spore-formers

Organism	Missing genes
***Bacilli***	
*Bacillus selenitireducens*	*sigE, sigF, sigG, sigK, spmA, spmB, spoIID, spoIIE, spoIIGA, spoIIM, spoIIP, spoIIR, spoIIAA-spoIIIAH, spoIVA, spoIVB, spoIVFA, spoIVFB,* any SASP genes
*Exiguobacterium sibiricum* 255-15, *Exiguobacterium* sp. At1b	Same as above
*Macrococcus caseolyticus*	Same as above
***Clostridia***	
*Acetohalobium arabaticum*	*bofA, gerM, sbcC, sleC, sleL, yhaX, yisY,*
*Ammonifex degensii*	ftsA, spoQ^*^, spoIVFB, spoVG, cotF, yusN
*Caldicellulosiruptor* spp.	*ftsA, spoIIM* (some), *spoIIIAB* (some), *spoIIIAF, spoVK, spoVR, yabQ, yyaC*
*Ethanoligenens harbinense* YUAN-3	*spoIIM, spoIIP, spoIIIAF, spoVE, etfA*
*Eubacterium rectale*	*sigF, spmA, spmB, spoIIAA, spoIIM, spoIIIAB, spoIIIAF, yabQ*
*Halanaerobium hydrogeniformans*	Any SASP genes
*Ruminococcus albus* 7	*spmA, spmB, spoIIM, spoIIR, spoIIIAB, spoIIIAF, spoIIIE, spoVE, yabQ, yqfC*
*Syntrophothermus lipocalidus* DSM 12680	*spoVG, sleB, cwlJ*
*Clostridiales* genomosp. BVAB3 str. UPII9-5	Any SASP genes

Using phylogenetic profiles to explain asporogenous phenotypes requires certain caution. Thus, the initial proteome of *Clostridium tetani* E88, an asporogenous mutant used as a vaccine strain, had no *minE*, *spoIIIAC*, *spoIIID*, *spoVG*, *spoVM*, *spoVS*, *ssp4*, *abrB*, *bofA*, *yabP*, *yabQ* or *yqfC* gene products (see GenBank entry AE015927.1). However, tblastn searches of *C. tetani* genome sequence allowed identification of all these genes, as well as of the gene for the C-terminal part of SigK (Tables 2 and S3). These (mostly short) ORFs were not translated in the original annotation (Brüggemann *et al*., 2003) and were missed in the subsequent comparative analysis of clostridial sporulation (fig. 2 of Paredes *et al*., 2005). In the end, it appears that *C. tetani* E88 has all (known) essential sporulation genes and its asporogenous phenotype results either from its inability to properly process pro-σ^K^, or, as suggested by Paredes and colleagues (2005), from defects in the sporulation signal processing machinery, or, as discussed above, from some point mutations in essential sporulation genes.

### Sporulation genes of uncultured clostridia

In the end of 2011, when this manuscript was in preparation, Japanese scientists released three complete genomes of unculturable segmented filamentous bacteria *Cand.* Arthromitus spp., isolated from rat and mouse intestines (Kuwahara *et al*., 2011; Prakash *et al*., 2011). A detailed description of a draft genome of the mouse strain, assembled into five contigs, has also been published (Sczesnak *et al*., 2011). Despite their highly reduced genomes (∼ 1.6 Mb) and the absence of cultured representatives, *Cand*. Arthromitus spp. have been long known to form mature spores (Chase and Erlandsen, 1976; Kuwahara *et al*., 2011). Thus, availability of these genomes offered an excellent possibility of testing the key conclusions of this work against a genuine near-minimal set of sporulation genes. As noted in the genome descriptions, *Cand*. Arthromitus spp. encode at least 66 sporulation genes (see Fig. 1), including all apparently essential ones [Table 3, see also table S4 in references (Kuwahara *et al*., 2011) and (Sczesnak *et al*., 2011)]. At the same time, *Cand*. Arthromitus spp. lack most of the genes that appeared dispensable based on the analysis of other genomes, such as *spoIVFA*, *spoVAA, spoVAB, spoVAEA, spoVAF, spoVK, spoVR, bofA and bofC* (Table S3). The sporulation gene set of *Cand*. Arthromitus spp. supports an extremely streamlined control mechanism for regulating sporulation gene expression that includes the four sigma factors, Spo0A, SpoIIAA, SpoIIAB, SpoIIE, SpoIIGA, SpoIIR, SpoIIIA proteins, SpoIIID, SpoIIIJ and SpoIVB (see Table S3 and fig. S2B in Kuwahara *et al*., 2011). *Candidatus* Arthromitus spp. also encode engulfment proteins SpoIID, SpoIM and SpoIIP, and the putative ‘clostridial SpoIIQ’ of the CD0125 family (see above); spore cortex biosynthesis proteins YabP, YabQ, YlbJ, YqfC and YqfD; spore cortex-lytic enzyme SleC and *N*-acetylmuramoyl-l-alanine amidases CwlA, CwlC and CwlD (Table S2). In keeping with their reduced genome sizes, each *Cand*. Arthromitus sp. carries just two SASP genes, a single *gerABC* operon, no *gerD* or *gerP* genes, and a greatly reduced set of spore coat proteins. Nevertheless, this streamlined sporulation gene set is evidently sufficient to guide formation of viable spores.

Another uncultured clostridium with a fully sequenced genome, *Clostridiales* genomospecies BVAB3, has been detected by PCR in several cases of recurrent bacterial vaginosis (Fredricks *et al*., 2005). Despite having a larger genome (1810 kb) than *Cand*. Arthromitus spp., this organism lacks most sporulation genes (Fig. 1) and can be safely assumed to be asporogenous. Therefore, an ability of these bacteria to survive antibiotic treatments by forming spores does not seem to be a plausible explanation for the high incidence of recurrent vaginosis in the carriers of BVAB3 (Marrazzo *et al*., 2008).

## Discussion

The ability to form endospores is a key distinguishing trait of many genera in the *Firmicute*s phylum. With more than 12% of all *B. subtilis* genes expressed primarily during sporulation, it is a major event in the cell development that also affects other processes, including, for example, production of insecticidal crystal toxins in *Bacillus thuringiensis* and solventogenesis in *C. acetobutylicum* (Schnepf *et al*., 1998; Paredes *et al*., 2005). Obviously, only a relatively small fraction of sporulation-related genes are truly indispensable: mutations in most recently identified σ^F^- and σ^G^-regulated genes did not cause any sporulation defects (Eichenberger *et al*., 2003; 2004; Wang *et al*., 2006), suggesting that most essential sporulation genes had already been identified earlier (and assigned *spo* names, from *spo0* to *spoVI*). Nevertheless, even some *spo* genes appeared dispensable in certain genetic backgrounds, whereas others, while essential for sporulation, had clear orthologues in non-sporulating bacteria (Onyenwoke *et al*., 2004; Rigden and Galperin, 2008). As a result, despite the very useful compilations by Stragier (2002), Wiegel and colleagues (Onyenwoke *et al*., 2004) and other researchers, there still does not seem to be a widely recognized standard list of essential sporulation genes. This situation has been further illuminated by the recent controversy regarding sporulation in *Mycobacterium marinum*, with one group finding apparent mycobacterial orthologues for several sporulation genes (Ghosh *et al*., 2009) and the other group arguing (correctly) that all those genes are present in many non-endospore-forming species and have functions not exclusive for sporulation (Traag *et al*., 2010). Further, recent studies of Spo0A phosphorylation in clostridia (Worner *et al*., 2006; Underwood *et al*., 2009; Steiner *et al*., 2011) and characterization of sporulation-related genes in *C. acetobutylicum* (Alsaker and Papoutsakis, 2005; Jones *et al*., 2008) and of the spore proteome of *C. difficile* (Lawley *et al*., 2009) demonstrated that, despite conservation of the core sporulation machinery in both bacilli and clostridia, there are clear differences between the two groups. Thus, the goal of this work was to use the treasure trove of completely sequenced firmicute genomes to trace the presence or absence of (known) sporulation genes among spore-forming and asporogenous bacteria and use these patterns to define a minimal set of sporulation-specific genes in bacilli and clostridia.

Surprisingly, even the initial task of separating the species with completely sequenced genomes into spore-forming and asporogenous bacteria proved to be fairly complicated. Our analysis of the likely ‘sporulation core’ showed that very few sporulation genes are conserved in all spore-formers (Table 3). Many widely conserved sporulation genes turned out to be non-essential, such as, for example, the *dpaAB* genes that are missing in several spore-forming clostridia (Table S1) with their function apparently taken over by the electron transfer flavoprotein EtfA (Onyenwoke *et al*., 2004; Orsburn *et al*., 2010). On the other hand, previous studies observed widespread phylogenetic distribution, both within and outside of the phylum *Firmicutes*, of many supposedly sporulation-specific genes, including *cotJC*, *cotH*, *cotSA*, *spo0M*, *spoIIM*, *spoVG*, *spoVR*, *spoVS*, *gerAB*, *gerM*, *smpA* and *smpB* (Onyenwoke *et al*., 2004; Wu *et al*., 2005; Rigden and Galperin, 2008). In the end, we had to rely on the initial microbiological descriptions of the sequenced strains, where available, or the corresponding species or genera. In most cases, these descriptions contained at least some indications as to the (in)ability of the respective organisms to form spores. For some organisms, sporulation data were simply not available. As an example, *Thermincola potens* strain JR has been isolated based on its ability to effectively couple oxidation of acetate to the reduction of iron electrodes (Wrighton *et al*., 2008) and assigned to the genus *Thermincola* based on 99% identity of its 16S rRNA sequence with the previously described *Thermincola carboxydophila* and *Thermincola ferriacetica*. Its genome was then sequenced without any microbiological characterization of the organism (Byrne-Bailey *et al*., 2010). Since *T. ferriacetica* forms spores (Zavarzina *et al*., 2007), whereas *T. carboxydophila* has not been seen to do that so far (Sokolova *et al*., 2005), there was no easy way to predict whether *T. potens* strain JR is spore-forming. In contrast, all organisms listed in our Table S2 have been at some point reported to be asporogenous even though some of them carry large numbers of sporulation-related genes (Fig. 1) and there remains a distinct possibility that proper conditions for their sporulation have not yet been found.

These observations suggest that the asporogenous phenotype could depend on the absence (or a mutation) of a single gene, which would be hard to recognize from the phylogenetic profiles. The ability to form spores is easily lost even within spore-forming genera, as it happened, for example, in *B. selenitireducens, C. sticklandii* and *C. tetani* E88 (Switzer Blum *et al*., 1998; Brüggemann *et al*., 2003; Fonknechten *et al*., 2010). Therefore, one should not necessarily assume that the sequenced genome of a normally spore-forming species contains the full set of functional (i.e. non-frameshifted) sporulation genes. On the other hand, the loss of a substantial fraction of such genes, such as the one described above for *L. sphaericus* or for *Caldicellulosiruptor* spp. (see Table S3) should prevent sporulation of the respective organisms. A full understanding of what constitutes a minimal set of sporulation-specific genes would require a better understanding of the molecular functions of the encoded proteins.

As a complex developmental process, sporulation is tightly regulated. Accordingly, products of many widespread sporulation genes (Table 3) appear to have regulatory functions and participate in protein–DNA, protein–protein and/or protein–peptidoglycan interactions. In contrast to most metabolic processes, only a relatively small fraction of sporulation proteins seem to have an enzymatic activity (Table S3); some of them, like SpoIIQ, are former enzymes that have lost their activity. Therefore, assignment of a sporulation protein to a specific enzyme family should be taken with a grain of salt; many such proteins could have lost their enzymatic activity and retained only substrate (e.g. peptidoglycan) binding ability. In some cases, even when the initial activity has been preserved, it might not be directly relevant to the protein's role in sporulation. Thus, the potential catalase activity of the spore coat protein CotJC does not appear to be important for the assembly of the spore coat, whereas superoxide dismutase SodA, instead of its eponymous activity, appears to play a role in cross-linking of spore coat proteins (Henriques *et al*., 1998). Therefore, protein family-based assignments provided in Tables S3 and S4 should be considered only as tentative predictions in need of experimental verification. These assignments, coupled with the breadth of the phylogenetic distribution and high codon adaptation index (CAI) values, presented in Table S3, could be used to identify the most attractive targets for future experimental studies.

### Conclusions

This study demonstrates both the great potential and the inherent limitations of bioinformatics approaches to the characterization of complex systems, such as the sporulation machinery of *Bacilli* and *Clostridia*. While we can trace the patterns of presence and absence of certain genes across all available genomes (see Table S3 and the website http://www.ncbi.nlm.nih.gov/Complete_Genomes/Sporulation.html), suggest general enzymatic or peptidoglycan-binding functions for selected proteins and identify the likely cases of non-orthologous gene displacement, all these suggestions require experimental verification. Still, the current list of essential sporulation genes (Table 3) can be used as a foundation for categorization of the newly sequenced genomes into likely spore-forming, asporogenous or non-sporogenous. Future studies should establish the functions of the remaining uncharacterized genes and allow compiling the ultimate minimal set(s) of sporulation-specific genes in *Bacilli* and *Clostridia*.

## Experimental procedures

### Genomic data and sporulation gene lists

The complete genomic sequences and protein sets of firmicute species released before the end of 2011 (see Table S1) were extracted from the NCBI RefSeq database (Pruitt *et al*., 2012). The organisms were divided into non-spore-formers and potential spore-formers based on the presence in their genomes of the *spo0A*, *sspA* and *dpaAB* genes, followed by an analysis of the available literature, which identified 30 Spo0A-encoding non-spore-formers (Table S2). The initial set of *B. subtilis* sporulation genes was compiled as described previously (Rigden and Galperin, 2008), by combining the lists presented by Stragier and Losick (1996; Piggot and Losick, 2002; Errington, 2003; Onyenwoke *et al*., 2004). This set was supplemented by the sets of Spo0A-stimulated genes [categories I and II of Spo0A regulon members (Molle *et al*., 2003)], genes expressed under the control of sporulation-specific sigma factors σ^E^, σ^K^, σ^F^ and σ^G^ (Eichenberger *et al*., 2003; 2004; Steil *et al*., 2005; Wang *et al*., 2006), genes coding for the spore core and spore coat proteins (Driks, 2002), and the genes coding for exosporium proteins of *B. anthracis* (Redmond *et al*., 2004; Steichen *et al*., 2005). Redundant entries were removed by comparing the gene list against the 2009 release of the *B. subtilis* genome (Barbe *et al*., 2009) and the UniProt (The UniProt Consortium, 2011) entries for *B. subtilis* 168. The full list of *B. subtilis* genes (proteins) analysed in this study is provided in Table S3. Codon adaptation index values for sporulation proteins were taken from the Highly Expressed Genes Database (HEG-DB, (Puigbo *et al*., 2008b), where available, or calculated using the CAIcal server (Puigbo *et al*., 2008a). For the purposes of this work, a gene was considered essential for sporulation if a respective mutation (in a wild-type or a mutant background) resulted in a decrease in the number of viable heat-resistant spore by more than 1.5 logs (> 30-fold).

The sets of sporulation genes expressed in *C. difficile* was taken from the work of Lawley and colleagues (2009) and supplemented with a selection of *C. acetobutylicum* genes from Paredes and colleagues (2005), Jones and colleagues (2008) and Lawley and colleagues (2009). These genes were sorted by their COG assignments in the RefSeq database (Pruitt *et al*., 2012), where available. Known housekeeping genes and metabolic enzymes were removed from the set; orthologues of *B. subtilis* sporulation genes, already included in the list, were assigned to the respective COGs. The remaining genes were analysed for their phylogenetic distribution and those genes that were widely conserved among various clostridia have been added to the list of potential sporulation genes.

### Construction of sporulation COGs

Comparative analysis of the sporulation proteins from 122 Spo0A-encoding firmicute species released before 1 July 2011 (listed in Tables S1 and S3) was performed using a modification of the Clusters of Orthologous Groups of proteins (COG) approach (Tatusov *et al*., 1997; 2000), as described earlier (Mulkidjanian *et al*., 2006; Makarova *et al*., 2007). At the first step, 269 prokaryotic COGs (Tatusov *et al*., 2003) that already included sporulation proteins from *B. subtilis* or *C. acetobutylicum* were expanded by including proteins from newly sequenced genomes and, in some cases, subdivided into more specific clusters with fewer paralogues. The remaining firmicute proteins were compared against the existing set of 4872 prokaryotic COGs (Tatusov *et al*., 2003) using blastp with default parameters; proteins returning three or more best genome hits into the same COG were assigned to that COG. For the remaining sporulation proteins, 241 COGs were created anew manually, based on expert assessment of blast outputs for candidate proteins and their species-specific best hits, in a manner similar to the recently described protocol (Kristensen *et al*., 2010). The resulting protein clusters (COGs) were manually curated using the CODeditor software system (S. Smirnov, unpublished), specifically designed to streamline expert curation of the clustering data, splitting protein sequences into separate domains and analysis of the COG lists and their phylogenetic profiles. Phylogenetic patterns for small proteins and proteins that appeared to be missing in only one or two genomes were validated using the tblastn program (Altschul *et al*., 1997), as described previously (Natale *et al*., 2000). The previously not annotated predicted protein-coding genes identified with this approach were submitted to the RefSeq database (Pruitt *et al*., 2012).

### Protein annotation and taxonomic distribution

Sporulation proteins from *B. subtilis* and *C. acetobutylicum* were assigned to protein families in the Pfam (Punta *et al*., 2012), CDD (Marchler-Bauer *et al*., 2011), COG (Tatusov *et al*., 2003) or TIGRFAM (Selengut *et al*., 2007) databases by using CD-search (Marchler-Bauer and Bryant, 2004) against the CDD database (Marchler-Bauer *et al*., 2011). For distant similarity detection, uncharacterized protein sequences were subjected to comparisons of hidden Markov model family profiles against the Pfam and PDB (Rose *et al*., 2011) databases using HHsearch (Söding, 2005).

Identification of non-firmicute homologues of *B. subtilis* sporulation proteins was performed as described previously (Rigden and Galperin, 2008), based on the species lists in the Pfam, CDD and COG databases, where available, and verified using psi-blast (Altschul *et al*., 1997) searches. The blast hits were classified by phyla according to their assignments in the NCBI Taxonomy database (Federhen, 2012) and filtered to exclude hits from the *Firmicutes*.

## Supporting information

**Table S1.** Distribution of *spo0A*,*sspA* and *dpaAB* genes and the ability to form spores within the representatives of *Firmicutes* with completely sequenced genomes.

**Table S2.** Taxonomy of Spo0A-encoding non-spore-forming *Firmicutes*.

**Table S4.** Novel enzymatic function assignments for sporulation-related proteins.

**Table S3.** Distribution of known sporulationgenes among 122 Spo0A-encoding firmicute genomes. A constantly updated version of this file is being maintained at the website http://www.ncbi.nlm.nih.gov/Complete_Genomes/Sporulation.html.

**Fig. S1.** Correlation of spore formation andgenome size within the representatives of class (A) *Bacilli*and (B) *Clostridia.*

**Fig. S2.** Comparison of the phylograms builtfrom (A) concatenated sequences of 50 ribosomal proteins and (B) profiles of gene presence and absence from Table S3. Note theabsence in (B) of the non-sporulating *Macrococcus caseolyticus* and *Exiguobacterium* spp. (red outline) andthe shifted positions of *Lysinibacillus sphaericus* (green outline) and *Alicyclobacillus acidocaldarius* and *Kyrpidiatusciae* (blue outline).

**Fig. S3.** Amino acid substitutions in the active sites of peptidase M23-like (LytM) domains of SpoIIQ, CD0125 and SpoIVFA families.

**Fig. S4.** Sequence alignment of some known and newly translated SpoVM proteins.
