# Supplementary material for: Genomic determinants of sporulation in Bacilli and Clostridia: towards the minimal set of sporulation-specific genes
Source: Environ Microbiol. 2012 Nov;14(11):2870–90. doi: 10.1111/j.1462-2920.2012.02841.x (PMC3533761; doi:10.1111/j.1462-2920.2012.02841.x)
Supplement: Supplementary file 1 [file emi0014-2870-SD2.pdf]

Table S1. Firmicute genomes used in this work[a]

No.	Organism name	GenBank entry	Genome size, kb	*spo0A*	*sspA*	*dpaA*	*dpaB*	Sporu-lation	No. in Fig. 1	Spore COGs	Class	Order	Family
1	Alicyclobacillus acidocaldarius subsp. acido	CP001727	3,206	Y	Y	Y	Y	Y	102	Y	Bacilli	Bacillales	Alicyclobacillaceae
2	Alicyclobacillus acidocaldarius subsp. acido	CP002902	3,124	Y	Y	Y	Y	Y	93	N	Bacilli	Bacillales	Alicyclobacillaceae
3	Kyrpidia tusciae DSM 2912	CP002017	3,385	Y	Y	Y	Y	Y	109	Y	Bacilli	Bacillales	Alicyclobacillaceae
4	Anoxybacillus flavithermus WK1	CP000922	2,847	Y	Y	Y	Y	Y	143	Y	Bacilli	Bacillales	Bacillaceae
5	Bacillus amyloliquefaciens DSM 7	FN597644	3,980	Y	Y	Y	Y	Y	195	N	Bacilli	Bacillales	Bacillaceae
6	Bacillus amyloliquefaciens FZB42	CP000560	3,919	Y	Y	Y	Y	Y	191	Y	Bacilli	Bacillales	Bacillaceae
7	Bacillus amyloliquefaciens LL3	CP002634	3,995	Y	Y	Y	Y	Y	192	N	Bacilli	Bacillales	Bacillaceae
8	Bacillus amyloliquefaciens TA208	CP002627	3,938	Y	Y	Y	Y	Y	179	N	Bacilli	Bacillales	Bacillaceae
9	Bacillus amyloliquefaciens XH7	CP002927	3,939	Y	Y	Y	Y	Y	184	N	Bacilli	Bacillales	Bacillaceae
10	Bacillus anthracis str. A0248	CP001598	5,227	Y	Y	Y	Y	Y	184	Y	Bacilli	Bacillales	Bacillaceae
11	Bacillus anthracis str. Ames	AE016879	5,227	Y	Y	Y	Y	Y	174	Y	Bacilli	Bacillales	Bacillaceae
12	Bacillus anthracis str. 'Ames Ancestor'	AE017334	5,227	Y	Y	Y	Y	Y	178	Y	Bacilli	Bacillales	Bacillaceae
13	Bacillus anthracis str. CDC 684	CP001215	5,230	Y	Y	Y	Y	Y	193	Y	Bacilli	Bacillales	Bacillaceae
14	Bacillus anthracis str. Sterne	AE017225	5,229	Y	Y	Y	Y	Y	172	Y	Bacilli	Bacillales	Bacillaceae
15	Bacillus atrophaeus 1942	CP002207	4,168	Y	Y	Y	Y	Y	166	Y	Bacilli	Bacillales	Bacillaceae
16	Bacillus cellulosilyticus DSM 2522	CP002394	4,682	Y	Y	Y	Y	Y	156	Y	Bacilli	Bacillales	Bacillaceae
17	Bacillus cereus 03BB102	CP001407	5,270	Y	Y	Y	Y	Y	183	Y	Bacilli	Bacillales	Bacillaceae
18	Bacillus cereus AH187	CP001177	5,269	Y	Y	Y	Y	Y	200	Y	Bacilli	Bacillales	Bacillaceae
19	Bacillus cereus AH820	CP001283	5,303	Y	Y	Y	Y	Y	208	Y	Bacilli	Bacillales	Bacillaceae
20	Bacillus cereus ATCC 10987	AE017194	5,224	Y	Y	Y	Y	Y	193	Y	Bacilli	Bacillales	Bacillaceae
21	Bacillus cereus ATCC 14579	AE016877	5,412	Y	Y	Y	Y	Y	181	Y	Bacilli	Bacillales	Bacillaceae
22	Bacillus cereus B4264	CP001176	5,419	Y	Y	Y	Y	Y	196	Y	Bacilli	Bacillales	Bacillaceae
23	Bacillus cereus biovar anthracis str. CI	CP001746	5,196	Y	Y	Y	Y	Y	175	N	Bacilli	Bacillales	Bacillaceae
24	Bacillus cereus E33L	CP000001	5,301	Y	Y	Y	Y	Y	191	Y	Bacilli	Bacillales	Bacillaceae
25	Bacillus cereus F837/76	CP003188	5,288	Y	Y	Y	Y	Y	197	N	Bacilli	Bacillales	Bacillaceae
26	Bacillus cereus G9842	CP001186	5,387	Y	Y	Y	Y	Y	198	Y	Bacilli	Bacillales	Bacillaceae
27	Bacillus cereus Q1	CP000227	5,214	Y	Y	Y	Y	Y	203	Y	Bacilli	Bacillales	Bacillaceae
28	Bacillus clausii KSM-K16	AP006627	4,304	Y	Y	Y	Y	Y	147	Y	Bacilli	Bacillales	Bacillaceae
29	Bacillus coagulans 2-6	CP002472	3,073	Y	Y	Y	Y	Y	106	N	Bacilli	Bacillales	Bacillaceae
30	Bacillus coagulans 36D1	CP003056	3,552	Y	Y	Y	Y	Y	135	N	Bacilli	Bacillales	Bacillaceae
31	Bacillus cytotoxicus NVH 391-98	CP000764	4,087	Y	Y	Y	Y	Y	175	Y	Bacilli	Bacillales	Bacillaceae
32	Bacillus halodurans C-125	BA000004	4,202	Y	Y	Y	Y	Y	155	Y	Bacilli	Bacillales	Bacillaceae
33	Bacillus licheniformis ATCC 14580	AE017333	4,223	Y	Y	Y	Y	Y	186	Y	Bacilli	Bacillales	Bacillaceae
34	Bacillus licheniformis ATCC 14580	CP000002	4,223	Y	Y	Y	Y	Y	185	Y	Bacilli	Bacillales	Bacillaceae
35	Bacillus megaterium DSM 319	CP001982	5,097	Y	Y	Y	Y	Y	195	Y	Bacilli	Bacillales	Bacillaceae
36	Bacillus megaterium QM B1551	CP001983	5,097	Y	Y	Y	Y	Y		Y	Bacilli	Bacillales	Bacillaceae
37	Bacillus megaterium WSH-002	CP003017	4,984	Y	Y	Y	Y	Y	177	N	Bacilli	Bacillales	Bacillaceae
38	Bacillus pseudofirmus OF4	CP001878	3,859	Y	Y	Y	Y	Y	165	Y	Bacilli	Bacillales	Bacillaceae
39	Bacillus pumilus SAFR-032	CP000813	3,704	Y	Y	Y	Y	Y	209	Y	Bacilli	Bacillales	Bacillaceae
40	Bacillus subtilis BSn5	CP002468	4,094	Y	Y	Y	Y	Y	185	N	Bacilli	Bacillales	Bacillaceae
41	Bacillus subtilis subsp. natto BEST195	AP011541	4,097	Y	Y	Y	Y	Y	170	N	Bacilli	Bacillales	Bacillaceae
42	Bacillus subtilis subsp. spizizenii str. W23	CP002183	4,028	Y	Y	Y	Y	Y	179	Y	Bacilli	Bacillales	Bacillaceae
43	Bacillus subtilis subsp. spizizenii TU-B-10	CP002905	4,207	Y	Y	Y	Y	Y		N	Bacilli	Bacillales	Bacillaceae
44	Bacillus subtilis subsp. subtilis RO-NN-1	CP002906	4,012	Y	Y	Y	Y	Y		N	Bacilli	Bacillales	Bacillaceae
45	Bacillus subtilis subsp. subtilis str. 168	AL009126	4,216	Y	Y	Y	Y	Y		Y	Bacilli	Bacillales	Bacillaceae
46	Bacillus thuringiensis BMB171	CP001903	5,330	Y	Y	Y	Y	Y	179	Y	Bacilli	Bacillales	Bacillaceae
47	Bacillus thuringiensis serovar chinensis CT-	CP001907	5,487	Y	Y	Y	Y	Y	183	N	Bacilli	Bacillales	Bacillaceae
48	Bacillus thuringiensis serovar finitimus YBT-	CP002508	5,355	Y	Y	Y	Y	Y		N	Bacilli	Bacillales	Bacillaceae
49	Bacillus thuringiensis serovar konkukian str.	AE017355	5,238	Y	Y	Y	Y	Y	174	Y	Bacilli	Bacillales	Bacillaceae
50	Bacillus thuringiensis str. Al Hakam	CP000485	5,257	Y	Y	Y	Y	Y	164	Y	Bacilli	Bacillales	Bacillaceae
51	Bacillus weihenstephanensis KBAB4	CP000903	5,263	Y	Y	Y	Y	Y	205	Y	Bacilli	Bacillales	Bacillaceae
52	Geobacillus kaustophilus HTA426	BA000043	3,545	Y	Y	Y	Y	Y	150	Y	Bacilli	Bacillales	Bacillaceae
53	Geobacillus sp. C56-T3	CP002050	3,651	Y	Y	Y	Y	Y	149	Y	Bacilli	Bacillales	Bacillaceae

54	Geobacillus sp. WCH70	CP001638	3,465	Y	Y	Y	Y	Y	151	Y	Bacilli	Bacillales	Bacillaceae
55	Geobacillus sp. Y4.1MC1	CP002293	3,840	Y	Y	Y	Y	Y	137	Y	Bacilli	Bacillales	Bacillaceae
56	Geobacillus sp. Y412MC52	CP002442	3,629	Y	Y	Y	Y	Y	144	N	Bacilli	Bacillales	Bacillaceae
57	Geobacillus sp. Y412MC61	CP001795	3,668	Y	Y	Y	Y	Y	175	Y	Bacilli	Bacillales	Bacillaceae
58	Geobacillus thermodenitrificans NG80-2	CP000557	3,550	Y	Y	Y	Y	Y	152	Y	Bacilli	Bacillales	Bacillaceae
59	Geobacillus thermoglucosidasius C56-YS93	CP002835	3,893	Y	Y	Y	Y	Y	141	N	Bacilli	Bacillales	Bacillaceae
60	Geobacillus thermoleovorans CCB_US3_UF	CP003125	3,597	Y	Y	Y	Y	Y	151	N	Bacilli	Bacillales	Bacillaceae
61	Lysinibacillus sphaericus C3-41	CP000817	4,640	Y	Y	Y	Y	Y	116	Y	Bacilli	Bacillales	Bacillaceae
62	Oceanobacillus iheyensis HTE831	BA000028	3,631	Y	Y	Y	Y	Y	134	Y	Bacilli	Bacillales	Bacillaceae
63	Brevibacillus brevis NBRC 100599	AP008955	6,296	Y	Y	Y	Y	Y	179	Y	Bacilli	Bacillales	Paenibacillaceae
64	Paenibacillus mucilaginosus KNP414	CP002869	8,664	Y	Y	Y	Y	Y	181	N	Bacilli	Bacillales	Paenibacillaceae
65	Paenibacillus polymyxa E681	CP000154	5,395	Y	Y	Y	Y	Y	123	Y	Bacilli	Bacillales	Paenibacillaceae
66	Paenibacillus polymyxa M1	HE577054	6,231	Y	Y	Y	Y	Y	101	N	Bacilli	Bacillales	Paenibacillaceae
67	Paenibacillus polymyxa SC2	CP002213	5,732	Y	Y	Y	Y	Y	142	Y	Bacilli	Bacillales	Paenibacillaceae
68	Paenibacillus sp. JDR-2	CP001656	7,185	Y	Y	Y	Y	Y		Y	Bacilli	Bacillales	Paenibacillaceae
69	Paenibacillus sp. Y412MC10	CP001793	7,122	Y	Y	Y	Y	Y	157	Y	Bacilli	Bacillales	Paenibacillaceae
70	Paenibacillus terrae HPL-003	CP003107	6,083	Y	Y	Y	Y	Y	158	N	Bacilli	Bacillales	Paenibacillaceae
71	Alkaliphilus metalliredigens QYMF	CP000724	4,930	Y	Y	N	N	Y	126	Y	Clostridia	Clostridiales	Clostridiaceae
72	Alkaliphilus oremlandii OhILAs	CP000853	3,124	Y	Y	N	N	Y	102	Y	Clostridia	Clostridiales	Clostridiaceae
73	Clostridium acetobutylicum ATCC 824	AE001437	3,941	Y	Y	N	N	Y	112	Y	Clostridia	Clostridiales	Clostridiaceae
74	Clostridium acetobutylicum DSM 1731	CP002660	3,942	Y	Y	N	N	Y	109	N	Clostridia	Clostridiales	Clostridiaceae
75	Clostridium acetobutylicum EA 2018	CP002118	3,940	Y	Y	N	N	Y	109	N	Clostridia	Clostridiales	Clostridiaceae
76	Clostridium beijerinckii NCIMB 8052	CP000721	6,001	Y	Y	N	N	Y	105	Y	Clostridia	Clostridiales	Clostridiaceae
77	Clostridium botulinum A str. ATCC 19397	CP000726	3,863	Y	Y	N	N	Y	95	Y	Clostridia	Clostridiales	Clostridiaceae
78	Clostridium botulinum A str. ATCC 3502	AM412317	3,887	Y	Y	N	N	Y	103	Y	Clostridia	Clostridiales	Clostridiaceae
79	Clostridium botulinum A str. Hall	CP000727	3,761	Y	Y	N	N	Y	94	Y	Clostridia	Clostridiales	Clostridiaceae
80	Clostridium botulinum A2 str. Kyoto	CP001581	4,155	Y	Y	N	N	Y	104	Y	Clostridia	Clostridiales	Clostridiaceae
81	Clostridium botulinum A3 str. Loch Maree	CP000962	3,993	Y	Y	N	N	Y	102	Y	Clostridia	Clostridiales	Clostridiaceae
82	Clostridium botulinum B str. Eklund 17B	CP001056	3,800	Y	Y	N	N	Y	90	Y	Clostridia	Clostridiales	Clostridiaceae
83	Clostridium botulinum B1 str. Okra	CP000939	3,958	Y	Y	N	N	Y	99	Y	Clostridia	Clostridiales	Clostridiaceae
84	Clostridium botulinum Ba4 str. 657	CP001083	3,978	Y	Y	N	N	Y	109	Y	Clostridia	Clostridiales	Clostridiaceae
85	Clostridium botulinum BKT015925	CP002410	2,773	Y	Y	N	N	Y	86	N	Clostridia	Clostridiales	Clostridiaceae
86	Clostridium botulinum E3 str. Alaska E43	CP001078	3,660	Y	Y	N	N	Y	87	Y	Clostridia	Clostridiales	Clostridiaceae
87	Clostridium botulinum F str. 230613	CP002011	3,993	Y	Y	N	N	Y	99	N	Clostridia	Clostridiales	Clostridiaceae
88	Clostridium botulinum F str. Langeland	CP000728	3,995	Y	Y	N	N	Y	99	Y	Clostridia	Clostridiales	Clostridiaceae
89	Clostridium botulinum H04402 065	FR773526	3,920	Y	Y	N	N	Y	103	N	Clostridia	Clostridiales	Clostridiaceae
90	Clostridium cellulolyticum H10	CP001348	4,069	Y	Y	Y	Y	Y	94	Y	Clostridia	Clostridiales	Clostridiaceae
91	Clostridium cellulovorans 743B	CP002160	5,262	Y	Y	N	N	Y	106	Y	Clostridia	Clostridiales	Clostridiaceae
92	Clostridium clariflavum DSM 19732	CP003065	4,898	Y	Y	Y	Y	Y	95	N	Clostridia	Clostridiales	Clostridiaceae
93	Clostridium kluyveri DSM 555	CP000673	3,965	Y	Y	N	N	Y	104	Y	Clostridia	Clostridiales	Clostridiaceae
94	Clostridium kluyveri NBRC 12016	AP009049	3,896	Y	Y	N	N	Y	84	Y	Clostridia	Clostridiales	Clostridiaceae
95	Clostridium ljungdahlii DSM 13528	CP001666	4,630	Y	Y	N	N	Y	112	Y	Clostridia	Clostridiales	Clostridiaceae
96	Clostridium novyi NT	CP000382	2,548	Y	Y	N	N	Y	86	Y	Clostridia	Clostridiales	Clostridiaceae
97	Clostridium perfringens ATCC 13124	CP000246	3,257	Y	Y	N	N	Y	83	Y	Clostridia	Clostridiales	Clostridiaceae
98	Clostridium perfringens SM101	CP000312	2,897	Y	Y	N	N	Y	84	Y	Clostridia	Clostridiales	Clostridiaceae
99	Clostridium perfringens str. 13	BA000016	3,031	Y	Y	N	N	Y	91	Y	Clostridia	Clostridiales	Clostridiaceae
100	Clostridium phytofermentans ISDg	CP000885	4,848	Y	Y	Y	Y	Y	73	Y	Clostridia	Clostridiales	Clostridiaceae
101	Clostridium saccharolyticum WM1	CP002109	4,663	Y	Y	Y	Y	Y	66	Y	Clostridia	Clostridiales	Clostridiaceae
102	Clostridium thermocellum ATCC 27405	CP000568	3,843	Y	Y	Y	Y	Y	97	Y	Clostridia	Clostridiales	Clostridiaceae
103	Clostridium thermocellum DSM 1313	CP002416	3,562	Y	Y	Y	Y	Y	98	N	Clostridia	Clostridiales	Clostridiaceae
104	Sulfobacillus acidophilus DSM 10332	CP003179	3,473	Y	Y	Y	Y	Y	89	N	Clostridia	Clostridiales	Family XVII. Incertae Sed
105	Sulfobacillus acidophilus TPY	CP002901	3,551	Y	Y	Y	Y	Y	70	N	Clostridia	Clostridiales	Family XVII. Incertae Sed
106	Symbiobacterium thermophilum IAM 14863	AP006840	3,566	Y	Y	Y	Y	Y	103	Y	Clostridia	Clostridiales	Family XVIII. Incertae Sec
107	Cellulosilyticum (Clostridium) lentocellum DS	CP002582	4,714	Y	Y	Y	Y	Y	78	N	Clostridia	Clostridiales	Lachnospiraceae
108	Heliobacterium modesticaldum Ice1	CP000930	3,075	Y	Y	Y	Y	Y	99	Y	Clostridia	Clostridiales	Heliobacteriaceae
109	Candidatus Desulforudis audaxviator MP104	CP000860	2,349	Y	Y	Y	Y	Y	66	Y	Clostridia	Clostridiales	Peptococcaceae
110	Desulfitobacterium hafniense DCB-2	CP001336	5,279	Y	Y	Y	Y	Y	105	Y	Clostridia	Clostridiales	Peptococcaceae
111	Desulfitobacterium hafniense Y51	AP008230	5,728	Y	Y	Y	Y	Y	75	Y	Clostridia	Clostridiales	Peptococcaceae

112	Desulfosporosinus orientis DSM 765	CP003108	5,863	Y	Y	Y	Y	Y	109	N	Clostridia	Clostridiales	Peptococcaceae
113	Desulfotomaculum acetoxidans DSM 771	CP001720	4,546	Y	Y	Y	Y	Y	113	Y	Clostridia	Clostridiales	Peptococcaceae
114	Desulfotomaculum carboxydivorans CO-1-S	CP002736	2,892	Y	Y	Y	Y	Y	93	N	Clostridia	Clostridiales	Peptococcaceae
115	Desulfotomaculum kuznetsovii DSM 6115	CP002770	3,601	Y	Y	Y	Y	Y	94	N	Clostridia	Clostridiales	Peptococcaceae
116	Desulfotomaculum reducens MI-1	CP000612	3,608	Y	Y	Y	Y	Y	97	Y	Clostridia	Clostridiales	Peptococcaceae
117	Desulfotomaculum ruminis DSM 2154	CP002780	3,969	Y	Y	Y	Y	Y	110	N	Clostridia	Clostridiales	Peptococcaceae
118	Pelotomaculum thermopropionicum SI	AP009389	3,025	Y	Y	Y	Y	Y	69	Y	Clostridia	Clostridiales	Peptococcaceae
119	Syntrophobotulus glycolicus DSM 8271	CP002547	3,407	Y	Y	Y	Y	Y	83	Y	Clostridia	Clostridiales	Peptococcaceae
120	Thermincola potens JR	CP002028	3,157	Y	Y	Y	Y	?	93	Y	Clostridia	Clostridiales	Peptococcaceae
121	Clostridium difficile 630	AM180355	4,290	Y	Y	Y	Y	Y	66	Y	Clostridia	Clostridiales	Peptostreptococcaceae
122	Clostridium difficile CD196	FN538970	4,111	Y	Y	Y	Y	Y	67	Y	Clostridia	Clostridiales	Peptostreptococcaceae
123	Clostridium difficile R20291	FN545816	4,191	Y	Y	Y	Y	Y	67	Y	Clostridia	Clostridiales	Peptostreptococcaceae
124	Thermoanaerobacterium thermosaccharolyt	CP002171	2,786	Y	Y	Y	Y	?	93	Y	Clostridia	Thermoanaero	Family III. Incertae Sedis
125	Thermoanaerobacterium xylanolyticum LX-1	CP002739	2,534	Y	Y	Y	Y	Y	94	N	Clostridia	Thermoanaero	Family III. Incertae Sedis
126	Mahella australiensis 50-1 BON	CP002360	3,136	Y	Y	Y	Y	Y	85	N	Clostridia	Thermoanaero	Family IV.Incertae Sedis
127	Carboxydothermus hydrogenoformans Z-29	CP000141	2,402	Y	Y	Y	Y	Y	77	Y	Clostridia	Thermoanaero	Thermoanaerobacterace
128	Moorella thermoacetica ATCC 39073	CP000232	2,629	Y	Y	Y	Y	Y	65	Y	Clostridia	Thermoanaero	Thermoanaerobacterace
129	Tepidanaerobacter sp. Re1	CP002728	2,760	Y	Y	Y	Y	Y	78	N	Clostridia	Thermoanaero	Thermoanaerobacterace
130	Thermoanaerobacter brockii subsp. finnii Ak	CP002466	2,345	Y	Y	Y	Y	Y	83	Y	Clostridia	Thermoanaero	Thermoanaerobacterace
131	Thermoanaerobacter italicus Ab9	CP001936	2,451	Y	Y	Y	Y	Y	84	Y	Clostridia	Thermoanaero	Thermoanaerobacterace
132	Thermoanaerobacter mathranii subsp. math	CP002032	2,306	Y	Y	Y	Y	Y	82	Y	Clostridia	Thermoanaero	Thermoanaerobacterace
133	Thermoanaerobacter pseudethanolicus ATC	CP000924	2,363	Y	Y	Y	Y	Y	71	Y	Clostridia	Thermoanaero	Thermoanaerobacterace
134	Thermoanaerobacter sp. X513	CP002210	2,457	Y	Y	Y	Y	?	85	Y	Clostridia	Thermoanaero	Thermoanaerobacterace
135	Thermoanaerobacter sp. X514	CP000923	2,457	Y	Y	Y	Y	?	73	Y	Clostridia	Thermoanaero	Thermoanaerobacterace
136	Thermoanaerobacter tengcongensis MB4	AE008691	2,689	Y	Y	Y	Y	Y/N	94	Y	Clostridia	Thermoanaero	Thermoanaerobacterace
137	Thermoanaerobacter wiegelii Rt8.B1	CP002991	2,785	Y	Y	Y	Y	Y	90	N	Clostridia	Thermoanaero	Thermoanaerobacterace
138	Candidatus Arthromitus sp. SFB-mouse-Jap	AP012202	1,620	Y	Y	Y	Y	Y	63	N	Clostridia	Clostridiales	Clostridiaceae
139	Candidatus Arthromitus sp. SFB-mouse-Yit	AP012209	1,586	Y	Y	Y	Y	Y	61	N	Clostridia	Clostridiales	Clostridiaceae
140	Candidatus Arthromitus sp. SFB-rat-Yit	AP012210	1,516	Y	Y	Y	Y	Y	61	N	Clostridia	Clostridiales	Clostridiaceae

No.	Organism name	GenBank entry	Genome size, kb	*spo0A*	*sspA*	*dpaA*	*dpaB*	Sporu-lation	No. in Fig. 1	Spore COGs	Class	Order	Family
1	Clostridiales genomosp. BVAB3 str. UPII9-5	CP001850	1,810	Y	N	N	N	?	7	Y	Clostridia	Clostridiales	unclassified Clostridiales
2	Clostridium tetani E88	AE015927	2,799	Y	Y	N	N	N	84	Y	Clostridia	Clostridiales	Clostridiaceae
3	Eubacterium eligens ATCC 27750	CP001104	2,144	Y	Y	N	N	N	53	Y	Clostridia	Clostridiales	Eubacteriaceae
4	Eubacterium rectale ATCC 33656	CP001107	3,450	Y	Y	Y	Y	N	60	Y	Clostridia	Clostridiales	Eubacteriaceae
5	Thermaerobacter marianensis DSM 12885	CP002344	2,845	Y	Y	Y	Y	N	90	Y	Clostridia	Clostridiales	Family XVII. Incertae Sed
6	Roseburia hominis A2-183	CP003040	3,592	Y	Y	Y	Y	?	66	N	Clostridia	Clostridiales	Lachnospiraceae
7	Oscillibacter valericigenes Sjm18-20	AP012044	4,410	Y	Y	Y	Y	N	49	N	Clostridia	Clostridiales	Oscillospiraceae
8	Ethanoligenens harbinense YUAN-3	CP002400	3,009	Y	Y	Y	Y	N	63	Y	Clostridia	Clostridiales	Ruminococcaceae
9	Ruminococcus albus 7	CP002403	3,685	Y	Y	Y	Y	N	55	Y	Clostridia	Clostridiales	Ruminococcaceae
10	Syntrophomonas wolfei subsp. wolfei str. Gc	CP000448	2,936	Y	Y	Y	Y	N	71	Y	Clostridia	Clostridiales	Syntrophomonadaceae
11	Syntrophothermus lipocalidus DSM 12680	CP002048	2,406	Y	Y	Y	Y	N	79	Y	Clostridia	Clostridiales	Syntrophomonadaceae
12	Acetohalobium arabaticum DSM 5501	CP002105	2,470	Y	Y	Y	Y	N	95	Y	Clostridia	Halanaerobiale	Halobacteroidaceae
13	Halanaerobium hydrogeniformans	CP002304	2,613	Y	N	N	N	N	19	Y	Clostridia	Halanaerobiale	Halobacteroidaceae
14	Halanaerobium praevalens DSM 2228	CP002175	2,309	Y	N	N	N	N	24	N	Clostridia	Halanaerobiale	Halobacteroidaceae
15	Halothermothrix orenii H 168	CP001098	2,578	Y	Y	Y	Y	N	87	Y	Clostridia	Halanaerobiale	Halobacteroidaceae
16	Natranaerobius thermophilus JW/NM-WN-L	CP001034	3,166	Y	Y	Y	Y	N	91	Y	Clostridia	Natranaerobial	Natranaerobiaceae
17	Caldicellulosiruptor bescii DSM 6725	CP001393	2,920	Y	Y	Y	Y	N	69	Y	Clostridia	Thermoanaero	Family III Incertae Sedis
18	Caldicellulosiruptor hydrothermalis 108	CP002219	2,771	Y	Y	Y	Y	N	66	Y	Clostridia	Thermoanaero	Family III Incertae Sedis
19	Caldicellulosiruptor kristjanssonii 177R1B	CP002326	2,786	Y	Y	Y	Y	N	64	Y	Clostridia	Thermoanaero	Family III Incertae Sedis
20	Caldicellulosiruptor kronotskyensis 2002	CP002330	2,844	Y	Y	Y	Y	N	66	Y	Clostridia	Thermoanaero	Family III Incertae Sedis
21	Caldicellulosiruptor lactoaceticus 6A	CP003001	2,675	Y	Y	Y	Y	N	56	N	Clostridia	Thermoanaero	Family III Incertae Sedis
22	Caldicellulosiruptor obsidiansis OB47	CP002164	2,532	Y	Y	Y	Y	N	64	Y	Clostridia	Thermoanaero	Family III Incertae Sedis
23	Caldicellulosiruptor owensensis OL	CP002216	2,429	Y	Y	Y	Y	N	62	Y	Clostridia	Thermoanaero	Family III Incertae Sedis
24	Caldicellulosiruptor saccharolyticus DSM 89	CP000679	5,577	Y	Y	Y	Y	N	56	Y	Clostridia	Thermoanaero	Family III Incertae Sedis
25	Thermosediminibacter oceani DSM 16646	CP002131	2,280	Y	Y	Y	Y	N	86	Y	Clostridia	Thermoanaero	Family III. Incertae Sedis
26	Ammonifex degensii KC4	CP001785	2,129	Y	Y	Y	Y	N	68	Y	Clostridia	Thermoanaero	Thermoanaerobacterace

27	Bacillus selenitireducens MLS10	CP001791	3,592	Y	N	N	N	N	19	Y	Bacilli	Bacillales	Bacillaceae
28	Exiguobacterium sibiricum 255-15	CP001022	3,034	Y	N	N	N	N	22	Y	Bacilli	Bacillales	Family XII. Incertae Sedis
29	Exiguobacterium sp. AT1b	CP001615	3,000	Y	N	N	N	N	17	Y	Bacilli	Bacillales	Family XII. Incertae Sedis
30	Macrococcus caseolyticus JCSC5402	AP009484	2,102	Y	N	N	N	N	12	N	Bacilli	Bacillales	Staphylococcaceae

**No.**	**Organism name**	**GenBank entry**	**Genome size, kb**	***spo0A***	***sspA***	***dpaA***	***dpaB***	**Sporu-lation**	**No. in Fig. 1**	**Spore COGs**	**Class**	**Order**	**Family**
1	Butyrivibrio proteoclasticus B316	CP001810	4,405	N	N	N	N	N	11	N	Clostridia	Clostridiales	Lachnospiraceae
2	Eubacterium limosum KIST612	CP002273	4,317	N	N	N	N	N	15	N	Clostridia	Clostridiales	Eubacteriaceae
3	Lactobacillus plantarum WCFS1	AL935263	3,308	N	N	N	N	N	4	N	Bacilli	Lactobacillales	Lactobacillaceae
4	Enterococcus faecalis V583	AE016830	3,218	N	N	N	N	N	8	N	Bacilli	Lactobacillales	Enterococcaceae
5	Listeria innocua Clip11262	AL592022	3,011	N	N	N	N	N	11	N	Bacilli	Bacillales	Listeriaceae
6	Lactobacillus rhamnosus GG	FM179322	3,010	N	N	N	N	N	5	N	Bacilli	Lactobacillales	Lactobacillaceae
7	Listeria monocytogenes EGD-e	AL591824	2,945	N	N	N	N	N	9	N	Bacilli	Bacillales	Listeriaceae
8	Listeria ivanovii subsp. ivanovii PAM 55	FR687253	2,929	N	N	N	N	N	7	N	Bacilli	Bacillales	Listeriaceae
9	Lactobacillus casei ATCC 334	CP000423	2,895	N	N	N	N	N	4	N	Bacilli	Lactobacillales	Lactobacillaceae
10	Staphylococcus aureus subsp. aureus Mu5	BA000017	2,879	N	N	N	N	N	8	N	Bacilli	Bacillales	Staphylococcaceae
11	Clostridium sp. SY8519	AP012212	2,836	N	N	N	N	N	8	N	Clostridia	Clostridiales	Clostridiaceae
12	Listeria welshimeri serovar 6b str. SLCC533	AM263198	2,814	N	N	N	N	N	10	N	Bacilli	Bacillales	Listeriaceae
13	Listeria seeligeri serovar 1/2b str. SLCC395	FN557490	2,798	N	N	N	N	N	10	N	Bacilli	Bacillales	Listeriaceae
14	Clostridium sticklandii DSM 519	FP565809	2,715	N	N	N	N	N	11	N	Clostridia	Clostridiales	Peptostreptococcaceae
15	Staphylococcus haemolyticus JCSC1435	AP006716	2,685	N	N	N	N	N	11	N	Bacilli	Bacillales	Staphylococcaceae
16	Staphylococcus lugdunensis HKU09-01	CP001837	2,658	N	N	N	N	N	6	N	Bacilli	Bacillales	Staphylococcaceae
17	Carnobacterium sp. 17-4	CP002563	2,635	N	N	N	N	N	9	N	Bacilli	Lactobacillales	Carnobacteriaceae
18	Staphylococcus pseudintermedius HKU10-0	CP002439	2,617	N	N	N	N	N	8	N	Bacilli	Bacillales	Staphylococcaceae
19	Selenomonas sputigena ATCC 35185	CP002637	2,568	N	N	N	N	N	19	N	Negativicu	Selenomonada	Veillonellaceae
20	Staphylococcus carnosus subsp. carnosus T	AM295250	2,566	N	N	N	N	N	19	N	Bacilli	Bacillales	Staphylococcaceae
21	Tetragenococcus halophilus NBRC 12172	AP012046	2,563	N	N	N	N	N	3	N	Bacilli	Lactobacillales	Enterococcaceae
22	Staphylococcus saprophyticus subsp. sapro	AP008934	2,517	N	N	N	N	N	4	N	Bacilli	Bacillales	Staphylococcaceae
23	Lactobacillus buchneri NRRL B-30929	CP002652	2,506	N	N	N	N	N	5	N	Bacilli	Lactobacillales	Lactobacillaceae
24	Staphylococcus epidermidis ATCC 12228	AE015929	2,499	N	N	N	N	N	7	N	Bacilli	Bacillales	Staphylococcaceae
25	Acidaminococcus intestini RyC-MR95	CP003058	2,488	N	N	N	N	N	12	N	Negativicu	Selenomonada	Acidaminococcaceae
26	Megasphaera elsdenii strain DSM 20460	HE576794	2,475	N	N	N	N	N	12	N	Negativicu	Selenomonada	Veillonellaceae
27	Streptococcus sanguinis SK36	CP000387	2,388	N	N	N	N	N	4	N	Bacilli	Lactobacillales	Streptococcaceae
28	Lactococcus lactis subsp. lactis Il1403	AE005176	2,366	N	N	N	N	N	4	N	Bacilli	Lactobacillales	Streptococcaceae
29	Streptococcus gallolyticus UCN34	FN597254	2,351	N	N	N	N	N	4	N	Bacilli	Lactobacillales	Streptococcaceae
30	Acidaminococcus fermentans DSM 20731	CP001859	2,330	N	N	N	N	N	14	N	Negativicu	Selenomonada	Acidaminococcaceae
31	Lactobacillus brevis ATCC 367	CP000416	2,291	N	N	N	N	N	7	N	Bacilli	Lactobacillales	Lactobacillaceae
32	Streptococcus salivarius JIM8777	FR873482	2,211	N	N	N	N	N	4	N	Bacilli	Lactobacillales	Streptococcaceae
33	Streptococcus gordonii str. Challis substr. C	CP000725	2,197	N	N	N	N	N	5	N	Bacilli	Lactobacillales	Streptococcaceae
34	Streptococcus pseudopneumoniae IS7493	CP002925	2,191	N	N	N	N	N	7	N	Bacilli	Lactobacillales	Streptococcaceae
35	Streptococcus pneumoniae TIGR4	AE005672	2,161	N	N	N	N	N	8	N	Bacilli	Lactobacillales	Streptococcaceae
36	Streptococcus agalactiae 2603V/R	AE009948	2,160	N	N	N	N	N	4	N	Bacilli	Lactobacillales	Streptococcaceae
37	Streptococcus parasanguinis ATCC 15912	CP002843	2,154	N	N	N	N	N	5	N	Bacilli	Lactobacillales	Streptococcaceae
38	Streptococcus mitis B6	FN568063	2,147	N	N	N	N	N	6	N	Bacilli	Lactobacillales	Streptococcaceae
39	Streptococcus parauberis KCTC 11537	CP002471	2,144	N	N	N	N	N	4	N	Bacilli	Lactobacillales	Streptococcaceae
40	Veillonella parvula DSM 2008	CP001820	2,132	N	N	N	N	N	11	N	Negativicu	Selenomonada	Veillonellaceae
41	Lactobacillus kefiranofaciens ZW3	CP002764	2,113	N	N	N	N	N	5	N	Bacilli	Lactobacillales	Lactobacillaceae
42	Streptococcus dysgalactiae subsp. equisimil	AP010935	2,106	N	N	N	N	N	4	N	Bacilli	Lactobacillales	Streptococcaceae
43	Streptococcus pasteurianus ATCC 43144	AP012054	2,100	N	N	N	N	N	3	N	Bacilli	Lactobacillales	Streptococcaceae
44	Lactobacillus fermentum IFO 3956	AP008937	2,099	N	N	N	N	N	3	N	Bacilli	Lactobacillales	Lactobacillaceae
45	Streptococcus suis 05ZYH33	CP000407	2,096	N	N	N	N	N	4	N	Bacilli	Lactobacillales	Streptococcaceae
46	Aerococcus urinae ACS-120-V-Col10a	CP002512	2,081	N	N	N	N	N	8	N	Bacilli	Lactobacillales	Aerococcaceae
47	Lactobacillus helveticus DPC 4571	CP000517	2,081	N	N	N	N	N	5	N	Bacilli	Lactobacillales	Lactobacillaceae
48	Lactobacillus amylovorus GRL 1112	CP002338	2,068	N	N	N	N	N	5	N	Bacilli	Lactobacillales	Lactobacillaceae
49	Lactobacillus crispatus ST1	FN692037	2,043	N	N	N	N	N	3	N	Bacilli	Lactobacillales	Lactobacillaceae
50	Leuconostoc mesenteroides subsp. mesent	CP000414	2,038	N	N	N	N	N	3	N	Bacilli	Lactobacillales	Leuconostocaceae
51	Streptococcus mutans UA159	AE014133	2,033	N	N	N	N	N	9	N	Bacilli	Lactobacillales	Streptococcaceae

52	Streptococcus equi subsp. zooepidemicus M	CP001129	2,024	N	N	N	N	**N**	12	N	Bacilli	Lactobacillales	Streptococcaceae
53	Leuconostoc kimchii IMSNU 11154	CP001758	2,003	N	N	N	N	**N**	5	N	Bacilli	Lactobacillales	Leuconostocaceae
54	Lactobacillus reuteri DSM 20016	CP000705	2,000	N	N	N	N	**N**	6	N	Bacilli	Lactobacillales	Lactobacillaceae
55	Lactobacillus acidophilus NCFM	CP000033	1,994	N	N	N	N	**N**	7	N	Bacilli	Lactobacillales	Lactobacillaceae
56	Lactobacillus johnsonii NCC 533	AE017198	1,993	N	N	N	N	**N**	8	N	Bacilli	Lactobacillales	Lactobacillaceae
57	Streptococcus oralis Uo5	FR720602	1,959	N	N	N	N	**N**	4	N	Bacilli	Lactobacillales	Streptococcaceae
58	Leuconostoc gasicomitatum LMG 18811	FN822744	1,954	N	N	N	N	**N**	3	N	Bacilli	Lactobacillales	Leuconostocaceae
59	Lactococcus garvieae ATCC 49156	AP009332	1,950	N	N	N	N	**N**	3	N	Bacilli	Lactobacillales	Streptococcaceae
60	Filifactor alocis ATCC 35896	CP002390	1,931	N	N	N	N	**N**	15	N	Clostridia	Clostridiales	Peptostreptococcaceae
61	Thermodesulfobium narugense DSM 14796	CP002690	1,899	N	N	N	N	**N**	13	N	Clostridia	Thermoanaero	Thermodesulfobiaceae
62	Lactobacillus gasseri ATCC 33323	CP000413	1,894	N	N	N	N	**N**	4	N	Bacilli	Lactobacillales	Lactobacillaceae
63	Melissococcus plutonius ATCC 35311	AP012200	1,891	N	N	N	N	**N**	6	N	Bacilli	Lactobacillales	Enterococcaceae
64	Lactobacillus sakei subsp. sakei 23K	CR936503	1,885	N	N	N	N	**N**	5	N	Bacilli	Lactobacillales	Lactobacillaceae
65	Anaerococcus prevotii DSM 20548	CP001708	1,883	N	N	N	N	**N**	6	N	Clostridia	Clostridiales	Family XI. Incertae Sedis
66	Leuconostoc sp. C2	CP002898	1,877	N	N	N	N	**N**	5	N	Bacilli	Lactobacillales	Leuconostocaceae
67	Lactobacillus delbrueckii subsp. bulgaricus /	CR954253	1,865	N	N	N	N	**N**	4	N	Bacilli	Lactobacillales	Lactobacillaceae
68	Streptococcus pyogenes M1 GAS	AE004092	1,852	N	N	N	N	**N**	6	N	Bacilli	Lactobacillales	Streptococcaceae
69	Streptococcus uberis 0140J	AM946015	1,852	N	N	N	N	**N**	3	N	Bacilli	Lactobacillales	Streptococcaceae
70	Pediococcus pentosaceus ATCC 25745	CP000422	1,832	N	N	N	N	**N**	3	N	Bacilli	Lactobacillales	Lactobacillaceae
71	Pediococcus claussenii ATCC BAA-344	CP003137	1,829	N	N	N	N	**N**	4	N	Bacilli	Lactobacillales	Lactobacillaceae
72	Lactobacillus salivarius UCC118	CP000233	1,827	N	N	N	N	**N**	6	N	Bacilli	Lactobacillales	Lactobacillaceae
73	Finegoldia magna ATCC 29328	AP008971	1,798	N	N	N	N	**N**	8	N	Clostridia	Clostridiales	Family XI. Incertae Sedis
74	Leuconostoc citreum KM20	DQ489736	1,796	N	N	N	N	**N**	3	N	Bacilli	Lactobacillales	Leuconostocaceae
75	Streptococcus thermophilus CNRZ1066	CP000024	1,796	N	N	N	N	**N**	4	N	Bacilli	Lactobacillales	Streptococcaceae
76	Erysipelothrix rhusiopathiae str. Fujisawa	AP012027	1,788	N	N	N	N	**N**	6	N	Erysipelotr	Erysipelotricha	Erysipelotrichaceae
77	Oenococcus oeni PSU-1	CP000411	1,781	N	N	N	N	**N**	4	N	Bacilli	Lactobacillales	Leuconostocaceae
78	Coprothermobacter proteolyticus DSM 5265	CP001145	1,425	N	N	N	N	**N**	10	N	Clostridia	Thermoanaero	Thermodesulfobiaceae
79	Weissella koreensis KACC 15510	CP002899	1,422	N	N	N	N	**N**	4	N	Bacilli	Lactobacillales	Leuconostocaceae
80	Lactobacillus sanfranciscensis TMW 1.1304	CP002461	1,298	N	N	N	N	**N**	8	N	Bacilli	Lactobacillales	Lactobacillaceae

[a] The genomes are divided into three blocks: 140 known or likely spore-formers, 30 *spo0A*$^{+}$ non-spore-formers, and 80 *spo0A*$^{-}$ non-spore formers.
The first two blocks are sorted by taxonomy (Federhen, 2012); the third block is sorted in the decreasing order of the genome size.
Column K shows the number of "sporulation-related" genes plotted in Fig. 1.
Column L shows whether or not the respective genome has been included into the sporulation gene profiles (Table S3).

Table S2. Taxonomy of Spo0A-encoding non-spore-forming Firmicutes

Taxonomy	*spo0A* entry	*sspA* copies	*dpaAB* presence
**Class Bacilli**			
**Order Bacillales**			
**Family Bacillaceae**			
Bacillus selenitireducens MLS10	Bsel_2260	None	No
**Family Staphylococcaceae**			
Macrococcus caseolyticus JCSC5402	MCCL_1163	None	No
**Family XII. Incertae Sedis**			
Exiguobacterium sp. AT1b	EAT1b_0514	None	No
Exiguobacterium sibiricum 255-15	Exig_0912	None	No
**Class Clostridia**			
**Order Clostridiales**			
**Family Clostridiaceae**			
Clostridium tetani E88 (spo^{-} mutant strain)	CTC01569	2	No
**Family Eubacteriaceae**			
Eubacterium rectale ATCC 33656	EUBREC_2170	1	Yes
Eubacterium eligens ATCC 27750	EUBELI_01077	1	No
**Family Lachnospiraceae**			
Roseburia hominis A2-183	RHOM_08795	2	Yes
**Family Oscillospiraceae**			
Oscillibacter valericigenes Sjm18-20	OBV_15500	1	Yes
**Family Ruminococcaceae**			
Ethanoligenens harbinense YUAN-3	Ethha_0717	2	Yes
Ruminococcus albus 7	Rumal_0492	1	Yes
**Family Syntrophomonadaceae**			
Syntrophomonas wolfei str. Goettingen	Swol_0590	3	Yes
Syntrophothermus lipocalidus DSM 12680	Slip_0441	3	Yes
**Family XVII. Incertae Sedis**			
Thermaerobacter marianensis DSM 12885	Tmar_1157	6	Yes
**unclassified Clostridiales**			
Clostridiales genomosp. BVAB3 str. UPII9-5	ADC90814	None	No
**Order Halanaerobiales**			
**Family Halobacteroidaceae**			
Acetohalobium arabaticum DSM 5501	Acear_1727	3	Yes
Halanaerobium hydrogeniformans	Halsa_1385	None	No
Halanaerobium praevalens DSM 2228	Hprae_0900	None	No
Halothermothrix orenii H 168	Hore_06590	4	Yes
Natranaerobius thermophilus JW/NM-WN-LF	Nther_1689	3	Yes
**Order Thermoanaerobacterales**			
**Family Thermoanaerobacteraceae**			
Ammonifex degensii KC4	Adeg_1392	4	Yes
Thermosediminibacter oceani DSM 16646	Toce_1300	3	Yes

**Family III. Incertae Sedis**			
Caldicellulosiruptor bescii DSM 6725	Athe_1337	3	Yes
Caldicellulosiruptor hydrothermalis 108	Calhy_1389	3	Yes
Caldicellulosiruptor kristjanssonii 177R1B	Calkr_1324	3	Yes
Caldicellulosiruptor kronotskyensis 2002	Calkro_1364	3	Yes
Caldicellulosiruptor lactoaceticus 6A	Calla_0725	3	Yes
Caldicellulosiruptor obsidiansis OB47	COB47_1220	3	Yes
Caldicellulosiruptor owensensis OL	Calow_1129	3	Yes
Caldicellulosiruptor saccharolyticus DSM 8903	Csac_1900	3	Yes

The Spo0A locus tags (in blue) are hyperlinked to the protein entries in the RefSeq database

Table S4. Novel enzymatic function assignments for sporulation-related proteins

Gene	Protein entry (RefSeq, UniProt)	HHsearch distant homology results				Predicted role in sporulation
		Closest Pfam entry[a]	Functionally informative Pfam entry[a]	Closest functionally informative structure match: PDB code; protein name; activity[a]	Conservation of catalytic residues[b]	
cotH	16080659, Q45535	PF08757, CotH protein (287; 38%; 100%)	PF07804, HipA_C (41; 22%; 95%)	3akj; *Helicobacter pylori* CtkA; Ser/Thr kinase (157; 10%; 96%)	N160, N233; D179, D251	Signalling/regulation of spore coat formation
yhcO	255767212, P54599	PF07607, DUF1570 (103; 18%; 99%)	PF10460, Peptidase_M30 (205; 16%; 99%)	2y3u; *Clostridium histolyticum* collagenase G (185; 15%; 100%)	H523, H158; E524, E159; H527, H162; E555, E185	Protease, possible regulatory function
yngK	16078889, O35015	PF02638, DUF187 (358; 42%; 100%)	PF00128, α-amylase (165; 15%; 99%)	2xn2; *Lactobacillus acidophilus* α-galactosidase (223; 18%; 100%)	D482, D200; D552, D286	Carbohydrate degradation
yhbB	16077957, O31589	PF05382, Amidase_5 (124, 15%, 98%)	PF05382, Amidase_5 (124, 15%, 98%)	3pbi; *Mycobacterium tuberculosis* RipB; peptidoglycan hydrolase (95; 17%; 97%)	C152, C176; H201, H258; E213, N/A (see text)	Peptidoglycan breakdown
yndL	16078845, O31815	PF05908, DUF867 (190; 49%; 100%)	none	3a9l; Bacillus phage PHINIT1 poly-γ-glutamate hydrolase (201; 32%; 100%)	H40, H87; E45, E92; H103, H148	Cleavage of the γ-glutamyl peptide linkage of peptidoglycan or extracellular poly-γ–Glu

[a] - Values in brackets indicate: the length of alignment; sequence identity of alignment; HHsearch probability

[b] - Each pair shows the catalytic residue in structure match and the aligned residue in *B. subtilis* sequence
