# Supplementary material for: Genomic determinants of sporulation in Bacilli and Clostridia: towards the minimal set of sporulation-specific genes
Source: Environ Microbiol. 2012 Nov;14(11):2870–90. doi: 10.1111/j.1462-2920.2012.02841.x (PMC3533761; doi:10.1111/j.1462-2920.2012.02841.x)
Supplement: Supplementary file 3 [file emi0014-2870-SD1.pdf]

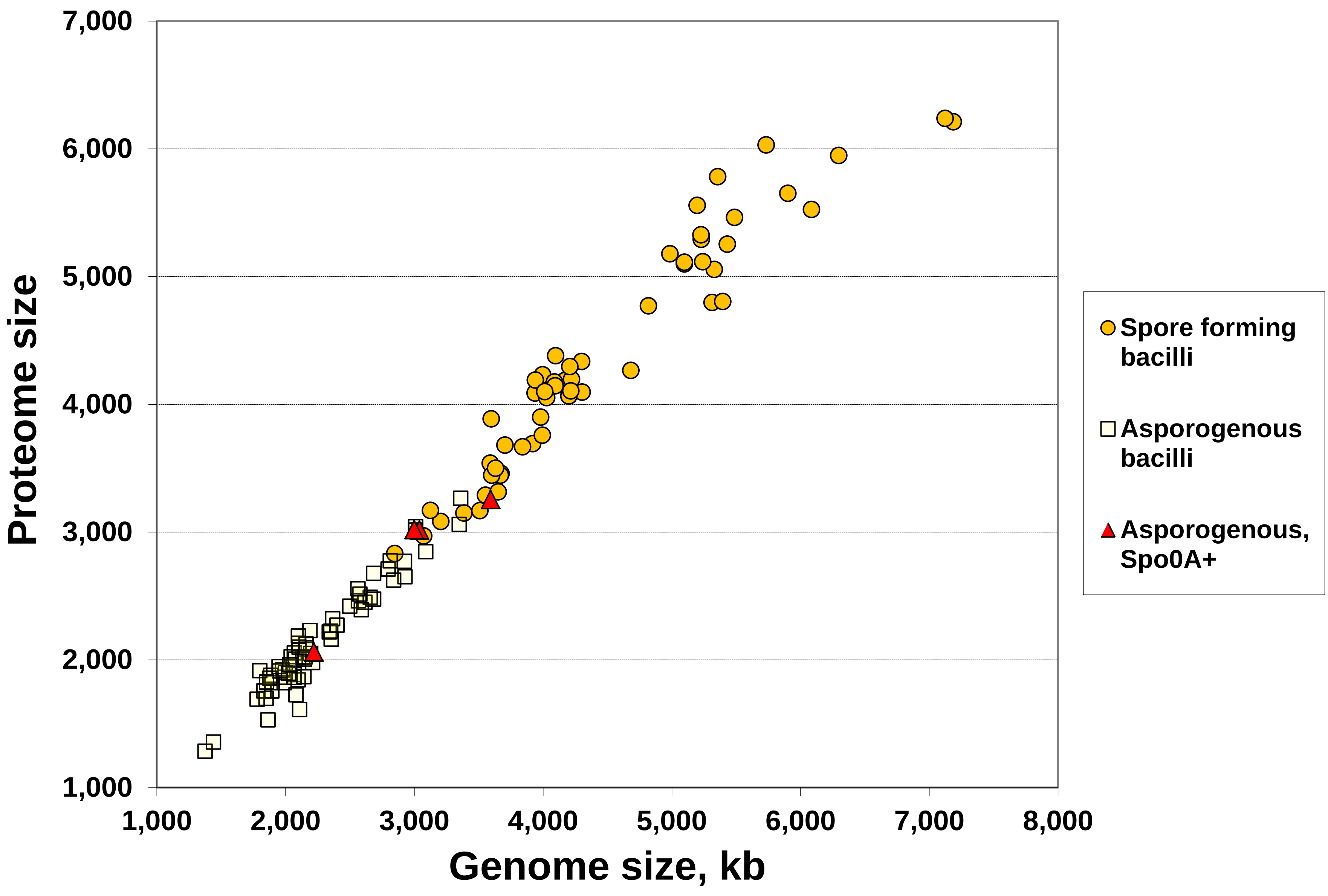

Proteome size
7,000
6,000
5,000
4,000
3,000
2,000
1,000
1,000
2,000
3,000
4,000
5,000
6,000
7,000
8,000
Genome size, kb
Spore forming bacilli
Asporogenous bacilli
Asporogenous, Spo0A+

Figure S1A

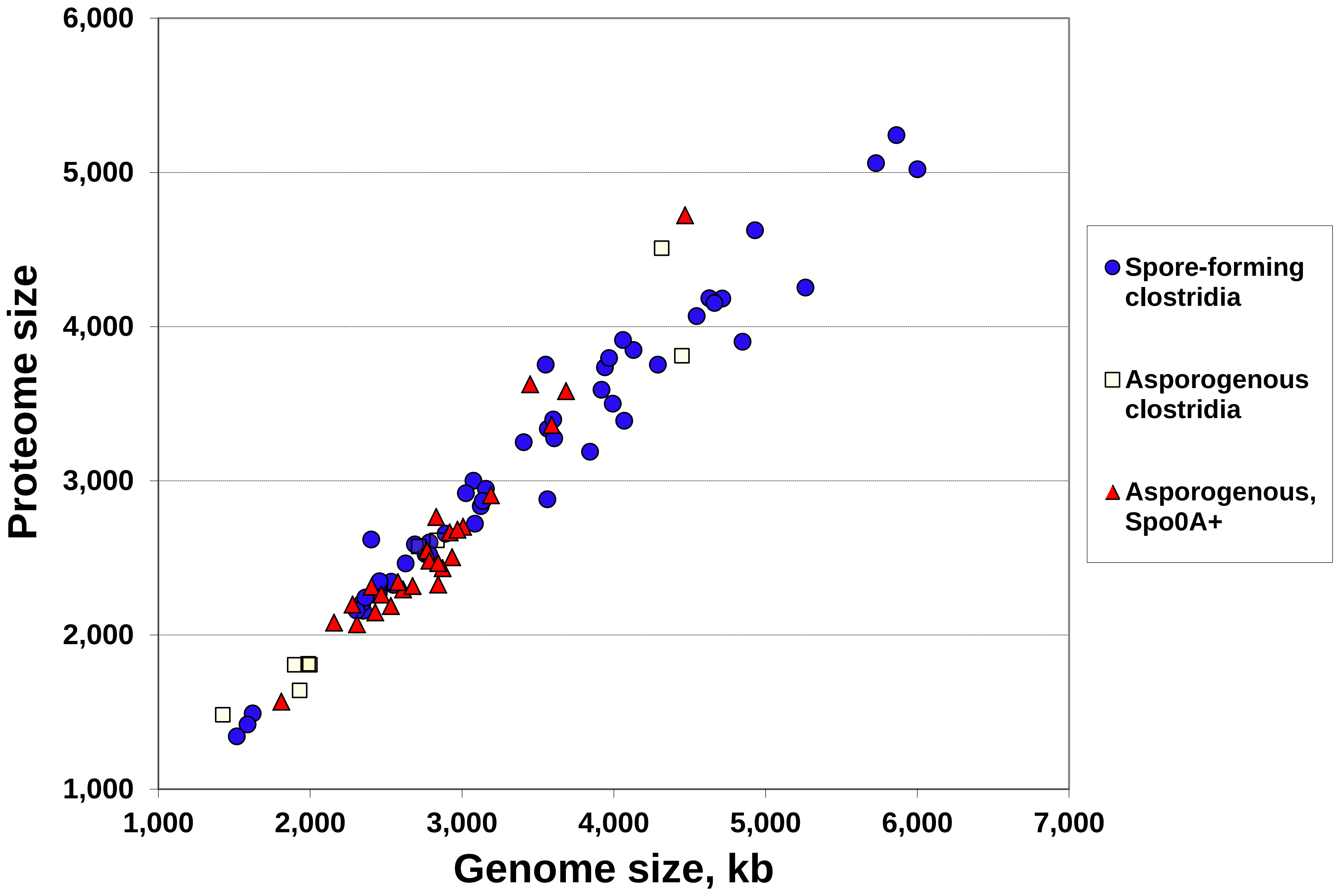
6,000
5,000
4,000
3,000
2,000
1,000
Proteome size
1,000
2,000
3,000
4,000
5,000
6,000
7,000
Genome size, kb
Spore-forming clostridia
Asporogenous clostridia
Asporogenous, Spo0A+

Figure S1B

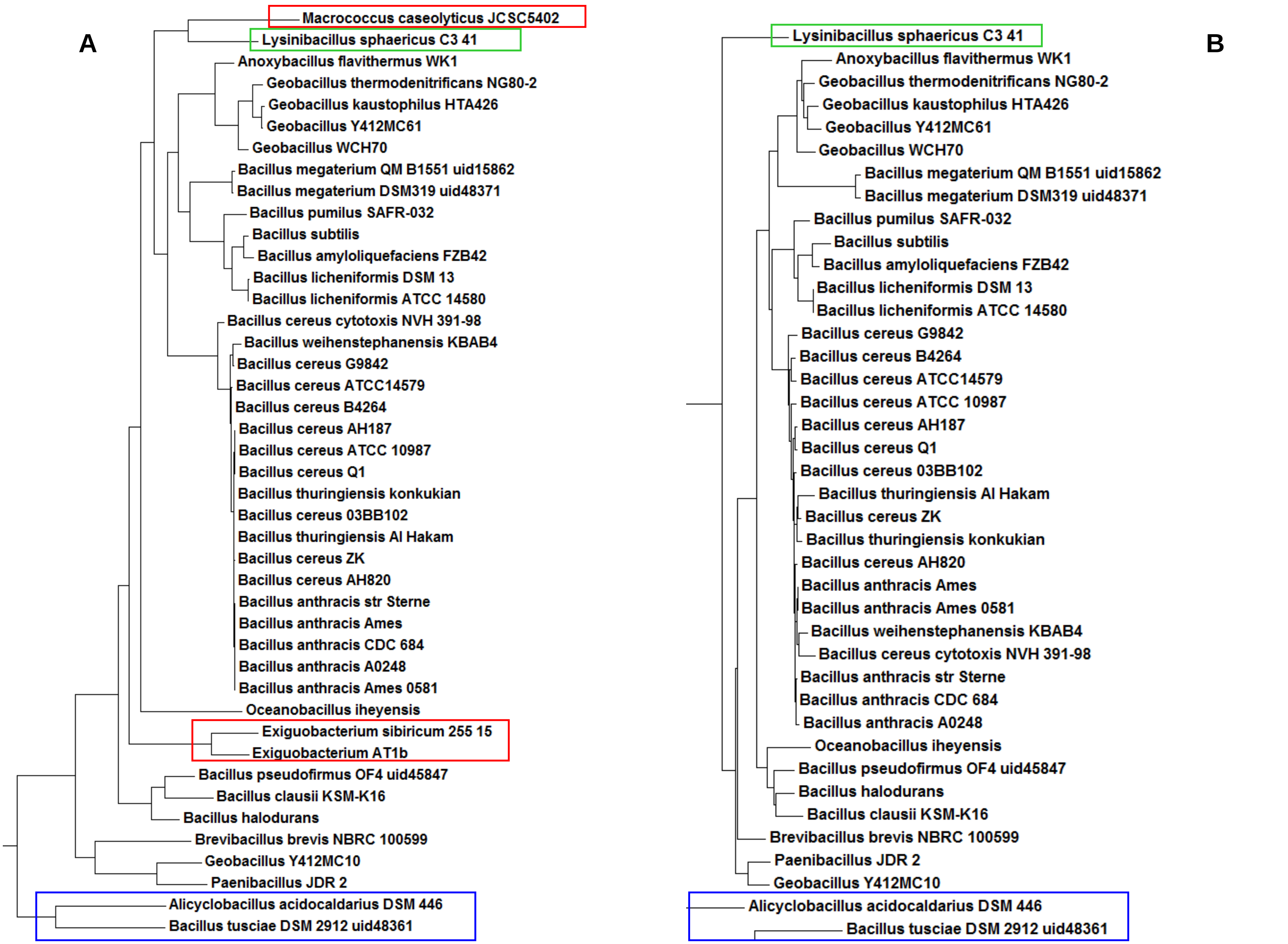

Figure S2. Comparison of the phylograms built from (A) concatenated sequences of 50 ribosomal proteins and (B) profiles of sporulation gene presence and absence from Table S3. Note the absence in panel B of the non-sporulating *Macrococcus caseolyticus* and *Exiguobacterium* spp. (red frames) and the shifted positions of *Lysinibacillus sphaericus* (green frame) and *Alicyclobacillus_acidocaldarius* and *Kyrpidia tusciae* (blue frame).

```
                       119 123                                          169                                                     204             LytM
SpoIIQ family           *   *                                            *                                                      * *          activity
Bsubt_16080708   117 SLSKGIDLAEKDGKD-5-SLSGTVVKAEK-DPVLGYVVEVEHAD-GLSTVYQSLS--EVSVEQGDKVKQNQVIGKSGKNLYSEDS-GNHVHFEI---RKDGVAMNP 216   No
Aflav_212640503  125 YPNKGVDIAMKNGES-5-SLSGTVVKAEK-DPLFGYVVHIQHDR-GVVTVYQSLK--DVQVKTGDTVKQGQVIAKAGTSEFNKEA-GVHVHFEI---RKDGQAVNP 224   No?
Oihey_23100416   114 YQSTGVDIAAANAET-5-AISGTVEEVKE-DPLLGNVVVLAHDE-DIKTYYASLE--EVSVSAGDKLTQGDKLGTAGKNIFGKDN-GTHVHFEI---RKSGAELNP 213   No
Ppoly_310644372  140 TANTGIDLSRSDDQA-5-ALSGKVSRVEQ-HPLVGNVVEITHDN-NLKTVYQSLS--DLKVKEGDQVKQNDVIASAGRNELEKDL-KTHLHFEV---YQDDKPVNP 239   No?
Ktusc_295697726  156 YPHHGIDLSAQNGKP-5-AAAGKVSKVED-DPLMGKVVEIAADN-GYTLYYASLA--DVSVKPGESVTQGQPIGTSGLNKFEAAD-KNHVHLEI---KKDGQDVNP 255   No?

SpoIVFA family
Bsubt_16079850   162 FQDNGEGIKVETSSD-3-VKEGYVVEVSK-DSQTGLTVKVQHAD-NTYSIYGELK--DVDVALYDFVDKGKKLGSIKLDDHNK----GVYYFAM---KDGDKFIDP 257   No
Aflav_212638536  143 FEHNGQGVVIETDQA-3-VEEGIVVFAGV-KEPYGKTVIIQHAD-GSETWYGKLS--AISVKLYDFIEAGKEVGKAEANNQK-----GVFYFAI---KQGDRFIDP 236   No
Oihey_23099503   153 FEANGIGIKIDPGQS-5-INEGVIVFAGN-DTSTEKTVKIQHSD-GSISQYGNLS--SVDVHLYQFIQANELVGTFTPTNEEQ----SVFFSIE---KD-NHYVDP 248   No
Ppoly_310643576  172 FVLSLKGVEITPNVA-9-IDAGRVLDVLN-HPKSGISVTIRHTG-GITATYGRLS--RSTLKVNDWVQEGEEIGVLPASHGGS--EAATLFLAI---QRGDQYIDP 274   No
Ktusc_295695345  189 YSSDHPWVVLDGVPG-5-AGKGVVEQVGR-RDPFGLYVVVNHGT-RGKTLYGSLG--DVVVKTGDYVYSGQSLGHLSAQRPAE------LLFGY---IVNGRYADP 283   No

CD0125 family
Cdiff_126697697  118 ETHKGVDISCTKGKEVKSLLNGIVVDVFD-DEEYGQSVKIKSDN-NIVVVYSNLDK-NVSVKKEQKVTEGQSLGTVGSTSQIESEEGIHVHLEA---YSGEKSIDP 217   Yes?
Cacet_15896114   140 TSIPGEYISPSKDSNVIAACDGVVKAID-----AGKVTISNDQT-GYTTVYDNLDEKSITVKPNDNVKQGQTIGKIGDSNYSN-4-TSCLYFEI-5-DGTYLAVDP 243   No?
Tbroc_320116872  175 TTHKGVDIGSNLGEPVLSAMDGIVTKVYK-DPKLGNTVVIKNGK--WETRYANLDD-EILAKEQEKIVRGQQIGKIGESAKFEVGEGPHLHFEL---LENGIPVDP 273   Yes?
CaArt_342732936  176 RTRDGVDLKADKGESVMSVLDGVVEKIDN-DLTEKGQYIVIKHDNGFKSVYTNLDE-EIKVVNGQKVRKGEVIATVGNSSGNY-5-GSHLNFVM---YLNNEEVNP 278   No?
Aorem_158321573  190 STHKGMDIKAKEGTPVKAALDGEVVEVLT-DKIDGITITLKHDN-DMYTRYSNLST-AVMVKVGDQIKKGQTISGVGKTASNKALEGPLLHFQV---LIDDKFVDP 289   Yes?

Pfam PF01551       1 RFHKGIDIAAPTGTPVYAAADGVVVFAGY-LGGYGNLVIIDHGN-GYETLYAHLS--KILVKVGQRVKAGQVIGTVGSTGRST---GPHLHFEI---RKNGKPVDP 96

3D structures
Staph_LytM_1QWY  183 GAHYGVDYAMPENSPVYSLTDGTVVQAGWSNYGGGNQVTIKEANSNNYQWYMHNN--RLTVSAGDKVKAGDQIAYSGSTGNST---APHVHFQR-4-IGNQYAVDP 284   Yes
Rumin_LytM_3NYY  123 RGHEGTDIXAEKNTP-7-XTDGVVTEKGW-LEKGGWRIGITAPT-GAYFYYAHLD-SYAELEKGDPVKAGDLLGYXGDSGYGE-7-PVHLHLGI-4-GTEEISVNP 234   Yes?
Paeru_pM23_2HSI  178 NPHSGLDFAVPAGTPIKAPAAGKVILIGD-YFFNGKTVFVDHGQ-GFISMFCHLS--KIDVKLGQQVPRGGVLGKVGATGRAT---GPHMHWNV---SLNDARVDP 273   Yes?
Vchol_pM23_2GU1  230 VPHNGTDFATPIGAPVYSTGDGKVIVVRK-HPYAGNYLVIEHNS-VYKTRYLHLD--KILVKKGQLVKRGQKIALAGATGRLT---GPHLHFEV-4-RPVDAMKAD 329   Yes?
Phi29_gp13_3CSQ  189 KGTLCIDFVGKTEKYPYYAPCDCTCVWRG-DAS--AYLAWTSD-12-TWVNVHES--PLPFDVGKKLKKGDLMGHTGIGGNVT---GDHWHFNV-4-EYQGWTKKP 296   No?
```

Supplementary Figure S3. Amino acid substitutions in the active sites of peptidase M23-like (LytM) domains of SpoIIQ, CD0125 and SpoIVFA families, compared to the St. aureus autolysin (PDB: 1QWY) and related structures. The sequences are from the following organisms: *Bacillus subtilis* subsp. *subtilis* str. 168, *Anoxybacillus flavithermus* WK1, *Oceanobacillus iheyensis* HTE831, *Paenibacillus polymyxa* SC2, and *Kyrpidia tusciae* DSM 2912 (SpoIIQ and SpoIVFA families) and from *Clostridium difficile* 630, *Clostridium acetobutylicum* ATCC 824, *Thermoanaerobacter brockii* subsp. *finnii* Ako-1, *Candidatus* Arthromitus sp. SFB-mouse-Japan, and *Alkaliphilus oremlandii* OhILAs (CD0125 family). The Zn^{2+}-binding (in white on red background) and phosphate-binding (in blue) residues of the peptidase M23 active site are from Firczuk *et al*., J. Mol. Biol. (2005) 354, 578–590. Residue numbering (top line) is based on *B. subtilis* SpoIIQ, prediction of peptidase activity is based solely on the conservation of the active-site residues. Protein gi's are hyperlinked to the respective entries in the NCBI protein database.

Previously annotated SpoVM proteins

```
B.subtilis_168_16078644    1655446 MKFYTIKLPKFLGGIVRAMLGSFRKD 1655526
B.amyloliquefac_154685997 1570528 MKFYTIKLPKFLGGIVRAMLGSFRKE 1570608
B.atrophaeus_311068102    1114122 MKFYTIKLPKFLGGIVRAMLGSFKKD 1114202
B.cellulosilyt_317129252  2749280 MKFYTIKLPKFIGGFVKACLGAFKKS 2749360
B.clausii_56964077        2435633 MKFYTIKLPKFLGGFVRAMLGSFKKE 2435713
B.halodurans_15615064     2619571 MKFYTIKLPKFLGGVVRAVLNSFKKG 2619651
B.licheniformis_52080184  1761281 MKFYTIKLPKFLGGIVRAMLGSFRKD 1761361
B.pseudofirmus_288553108  2360329 MKFYTIKLPKFLSGFVRAMLNSFKKG 2360409
B.pumilus_SAFR_157692261  1536437 MKFYTIKLPRFLGGIVRAMLGSFKKD 1536517
Anox.flavitherm_212639608 1769454 VKFYTIKLPKFLGGIVRAMLNSFKKG 1769534
Geo.kaustophilus_56419715 1201155 MKFYTIKLPRFLGGIVRAVLNAFKKG 1201235
Oceano.iheyensis_23098968 1557233 MKFYTIKLPKFVGGFVKVVIGIFKKD 1557313
Paenibacillus_251797731   4284117 MKFYTIKLPKFLGGFVKAILNTFQKN 4284197
```

New SpoVM annotations

```
Alicyclobacillus acidoc.  1383301 VKVYTIKLPRFLGGIVKAVLSTFTKD 1383378
Kyrpidia tusciae          1445197 VRFYTIKLPRFLGEIVKAILNAFHKD 1445274
B.anthracis_A0248         3674787 MRFYTIKLPKFLGGIVRAMLNTFKKD 3674710
B.anthracis_AmesAncestor  3674887 MRFYTIKLPKFLGGIVRAMLNTFKKD 3674810
B.atrophaeus_1942         1114122 MKFYTIKLPKFLGGIVRAMLGSFKKD 1114199
B.cereus_AH187            3662810 MRFYTIKLPKFLGGIVRAMLNTFKKD 3662733
B.cereus_AH820            3709652 MRFYTIKLPKFLGGIVRAMLNTFKKD 3709575
B.cereus_ATCC_10987       3650162 MRFYTIKLPKFLGGIVRAMLNTFKKD 3650085
B.cereus_ATCC_14579       3835599 MRFYTIKLPKFLGGIVRAMLNTFKKD 3835522
B.cereus_cytotoxis        2609645 MRFYTIKLPKFLGGIVRAVLNTFKKD 2609568
B.megaterium_DSM319       4076436 MKFYTIKLPKFLGGIVKVMLNSFKKD 4076359
B.thuringiensis_AlHakam   3704259 MRFYTIKLPKFLGGIVRAMLNTFKKD 3704182
B.thuringiensis_BMB171    3758743 MRFYTIKLPKFLGGIVRAMLNTFKKD 3758666
B.weihenstephanensis      3733672 MRFYTIKLPKFLGGIVRAMLNTFKKD 3733595
Lysinibacillus sphaericus 1591073 LKVYTFQLPKFVSSITRTCINLFKKD 1591150
Geo.thermodenitrificans   1102639 MKFYTIKLPKFLGGIVRAMLNAFRKG 1102716
Paenibacillus_sp_Y412MC10 2290947 MKFYTFKLPKFLGGFVKAILNTFHKS 2291024
Natranaerobius thermophil.1417452 MKFYTIKLPGFLAKLLRGFLGKGNKG 1417529
Clostridium novyi         1426462 MKIIAIKVPKFISKIIRLFKKDK--- 1426394
Clostr.acetobutylicum_824 1881169 MKIVAIKLPKFLSNIIKFFFRKKS-- 1881243
C.beijerinckii_8052       1373639 MKIIAFKLPKFLSNIIKFFRKKG--- 1373707
Clostr.perfringens_13     2011880 MRIMTIKLPKFLAKIVRMFKGNKKSD 2011803
Clostr.perfringens_SM101  1901228 MRIMAIKLPKFLAKIVRMFKGNKKSD 1901151
Clostr.thermocellum_27405 1556061 MKILVVKMPKFFGSILRKVLRMG--- 1555990
Caldicellulosiruptor_hydr. 614979 MRIMVFKMPRFLSKLIKKLFKISDDK  615074
Ammonifex_degensii        1382280 MRVIFIKVPGFLRFLLRRWFQAARGL 1382200
Symbiobacterium thermoph. 1504044 VRITVIRLPRPLGRMLMAIMRLFSGK 1504121
Cand. Arthromitus SFBmouse 658582 MKIIAIKLPKCLAKIVKIFKK-----  658644
```

Supplementary Figure S4. Sequence alignment of some known and newly translated SpoVM proteins. The numbers before and after the amino acid sequence indicate the genomic coordinates of the first and the last residue, respectively. The gi numbers of previously annotated proteins (in blue and bold) are hyperlinked to the respective RefSeq entries.
